# Antifungal Activities of Compounds Produced by Newly Isolated *Acrocarpospora* Strains

**DOI:** 10.3390/antibiotics12010095

**Published:** 2023-01-05

**Authors:** Ming-Jen Cheng, Jih-Jung Chen, Ming-Der Wu, Jyh-Yih Leu, Min Tseng

**Affiliations:** 1 Department of Life Science, Fu Jen Catholic University, New Taipei City 24205, Taiwan; 2 Department of Pharmacy, School of Pharmaceutical Sciences, National Yang Ming Chiao Tung University (NYCU), Taipei 112, Taiwan; 3 Department of Medical Research, China Medical University Hospital, China Medical University, Taichung 404, Taiwan; 4 Bioresource Collection and Research Center (BCRC), Food Industry Research and Development Institute (FIRDI), Hsinchu 300, Taiwan

**Keywords:** *Acrocarpospora punica* 04107M, Streptosporangiaceae, diterpenoid, antifungal activities

## Abstract

In our continued search for bioactive metabolites from cultures of rare *Actinobacteria* resources from all over Taiwan and various natural ecological environments, an active antimicrobial strain of *Acrocarpospora punica* 04107M was collected in Taitung County in Taiwan and prepared from soil. The bioassay-guided fractionation of the BuOH extract of a culture broth from *A*. *punica* 04107M led to the isolation of five previously undescribed compounds: Acrocarposporins A–E (Compounds **1**–**5**). All the constituents were confirmed by HRESIMS and 1D- and 2D-NMR spectroscopy. Their antifungal activity was also evaluated. Our results showed that four constituents (Compounds **1**, **2**, **4**, and **5**) possessed mild antifungal activity against *Aspergillus niger*, *Penicillium italicum*, *Candida albicans*, and *Saccharomyces cerevisiae*. It is worth mentioning that the chemical composition of *Acrocarpospora punica* 04107M has never been studied. This is the first report on diterpenoid metabolites from the genus *Acrocarpospora*.

## 1. Introduction

*Actinobacteria* are well known as excellent producers of prolific compounds with antimicrobial, insecticidal, and many other biological activities, and almost half of the active molecules discovered from natural sources belongs to this group. They are G(+), free-living saprophytic bacteria that are widely distributed in soil, water, and colonizing plants. Actinomycete inhabitants have been identified as one of the major groups in soil populations, which may vary by soil type. *Streptomycetes* produce a number of antibiotics and other bioactive compounds used in clinics. Their properties are prolific, and they can produce large quantities of antibiotics and various biologically active secondary metabolites [1,2,3,4,5,6,7]. However, the active metabolites of many Taiwanese neo-actinomycetes and their mechanisms of action remain unknown. It is necessary to study the active compounds from these new species of actinomycetes through scientific methods.

We recently isolated an unpublished novel strain, named 04107M, from the soil of Taitung County, which had a unique morphology and possessed antimicrobial activities as determined by our preliminary screening. This strain was determined to be *Acrocarpospora punica* 04107M based on its phenotypic and genotypic data.

The genus *Acrocarpospora* was first described by Tamura et al. [8] and is composed of the following three species: *A. corrugatum*, *A. macrocephala*, and *A. pleiomorpha* [8]. However, the microorganism discussed herein was isolated and identified by our research team. There have not been many chemical investigations of the genus *Acrocarpospora*, and only a few articles have reported on the classification of its molecular biology [9,10,11]. Currently, more than 300 microorganisms have been screened for in vitro antimicrobial activity and *A. punica *04107M was found to be one of the active ones. In the course of our continuous investigation of the biologically active metabolites present in Actinobacteria, we report herein the isolation of a new strain, *Acrocarpospora punica* 04107M (Figure 1), and the chemical investigation of the butanol extract of *A*. *punica* 04107M culture broths, concluding our investigation with the isolation of five new diterpenoids (Compounds **1**–**5**) (Figure 2) and an evaluation of their antifungal activity.

## 2. Results

### 2.1. Taxonomic Identification (Phenotypic and Genotypic Data) of Acrocarpospora punica 04107M

**Figure 1:**
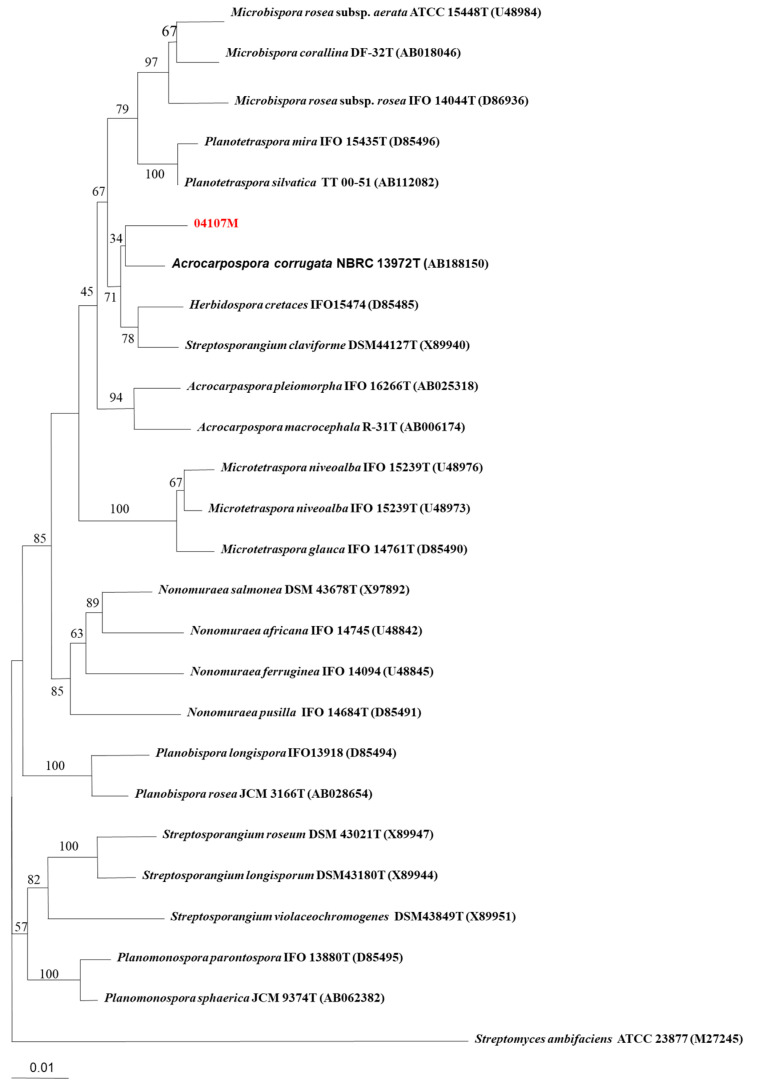
Neighbor-joining tree based on nearly complete 16S rDNA sequences showing the phylogenetic position of strain 04107M within the *Acrocarpospora* species. Numbers at the nodes indicate the percentage of 1000 bootstrap resampling procedures and only values over 50% are given. Bar: 0.01 substitutions per nucleotide position.

### 2.2. Structure Elucidation of Compounds

**Figure 2:**
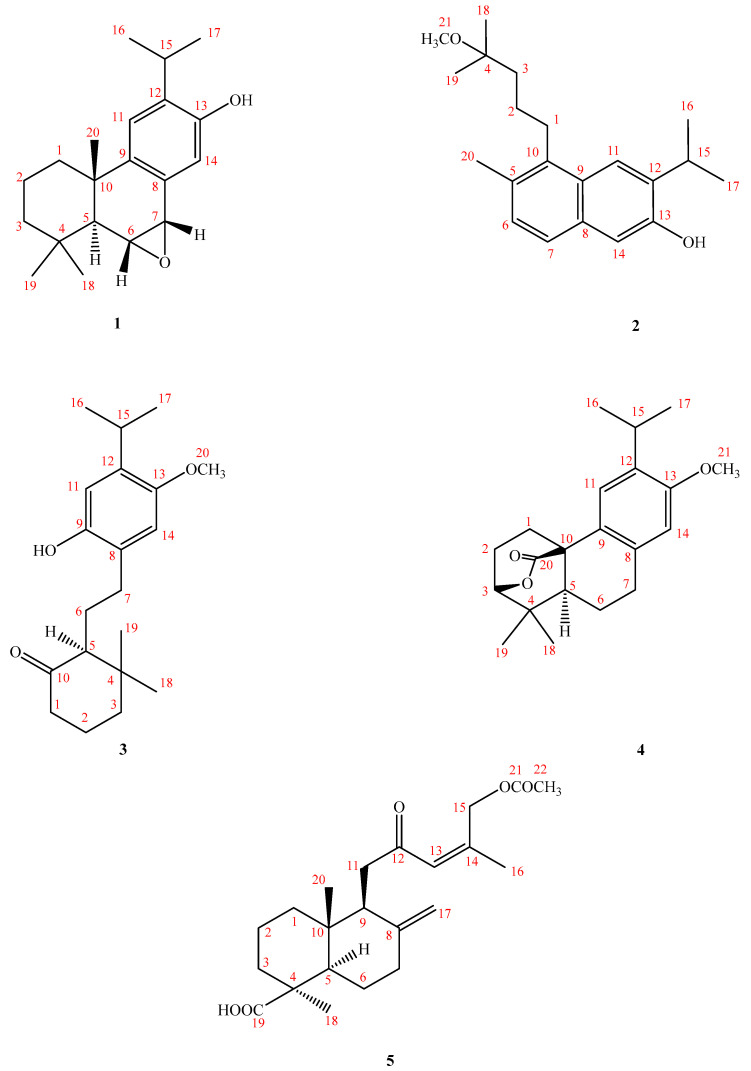
Compounds **1**–**5**, isolated from *Acrocarpospora punica* 04107M.

Compound **1** was isolated as a colorless oil with an [α]^24^_D_: -88.2 (*c* 0.01, CHCl_3_), and HRESIMS showed a molecular ion (M^+^) peak at *m/z* 300.2092 for C_20_H_28_O_2_, corresponding to seven indices of hydrogen deficiency (IHD). Analysis of its IR spectrum suggested that Compound **1** contained OH (3340 cm^−1^) and aromatic (1610 cm^−1^) and epoxide (1510 and 1233 cm^−1^) moieties. The ^1^H-NMR spectrum (see Supplementary Materials) revealed signals for an iPr group at δ_H_ 1.23 (6H, d, *J* = 7.4 Hz, and CH_3_-16/17) and 3.06 (1H, sep, *J* = 7.4 Hz, and H-15); three Me singlets at δ 1.29, 1.26, and 1.22 (each s, 3H); two oxymethines at δ_H_ 3.40 (dd, *J* = 10.8, 4.2 Hz, and H-6) and 5.18 (1H, br d, *J* = 4.2 Hz, and H-7); and a resonance at δ_H_ 2.28 (1H, br d, and *J* = 13.2 Hz) that was characteristic for the Hβ-1 signal of a dehydroabietane [12]. By comparing the ^1^H-NMR of Compound **1** with that of the known compound ferruginol [12], it was deduced that Compound **1** was indeed an abietane diterpene (Figure 2).

**Figure 3:**
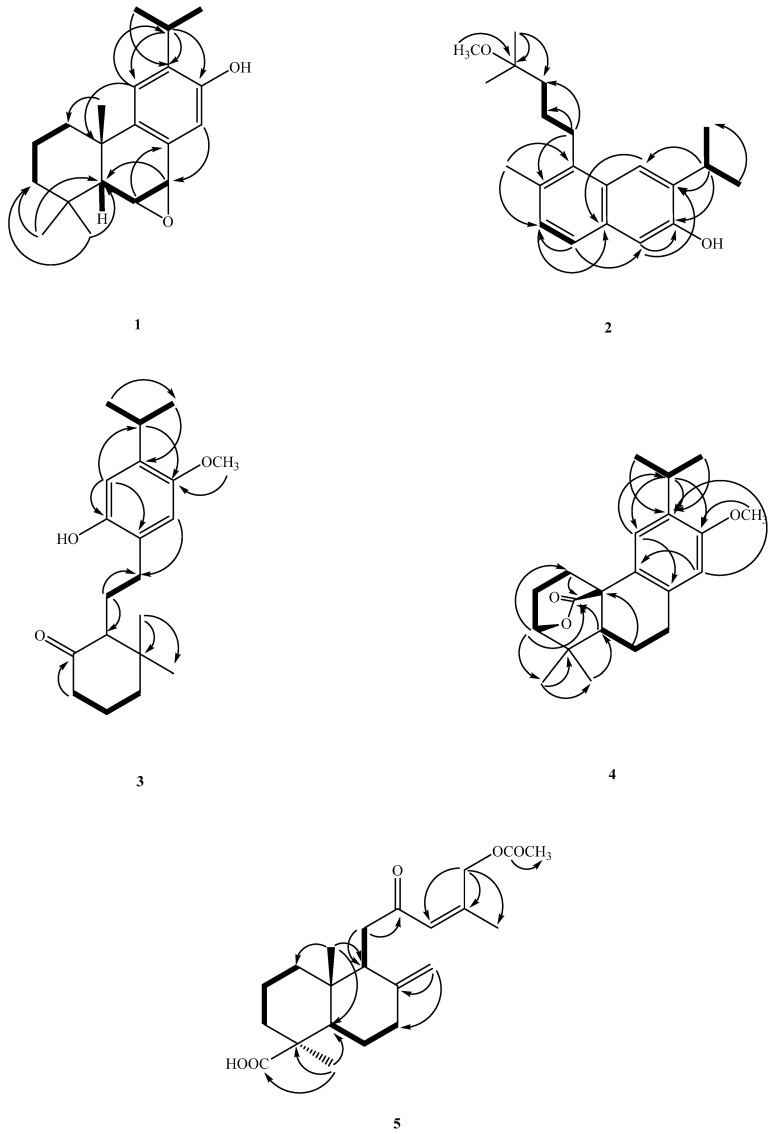
Key COSY (^1^H→^1^H) and HMBC (^1^H→^13^C) correlations of Compounds **1–5**.

**Figure 4:**
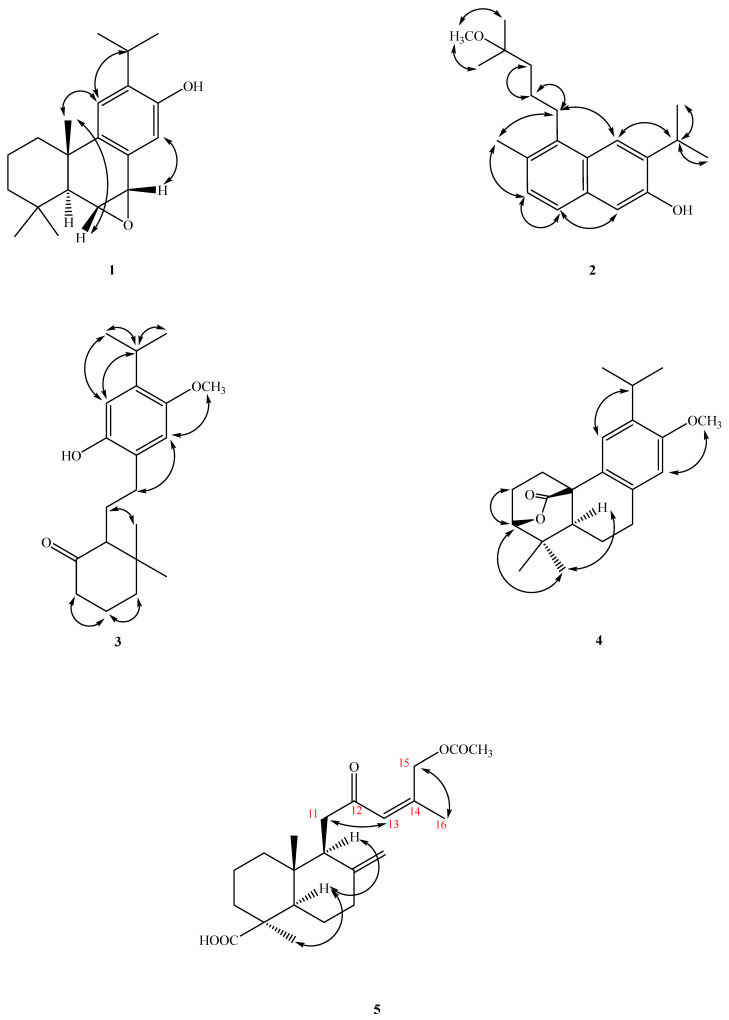
Key NOESY correlations (↔) of Compounds **1**–**5**.

Compound **1** was further confirmed to be a diterpene by the discovery of twenty ^13^C-NMR signals, with five methyls, three methylenes, six methines, and six quaternary carbons being present. The quaternary carbons could be further divided into one oxygen-bearing carbon at δ_H_ 153.5 (C-12) and three olefinic carbons at δ_C_ 149.1 (C-9), 132.8 (C-13), and 126.9 (C-8). An inspection of the ^13^C-NMR spectrum and the signals at δ_C_ 153.5, 149.1, 132.8, 126.9, 128.1 (C-14), and 109.1 (C-11) supported the presence of a tetrasubstituted benzene ring. Resonances at δ_C_ 76.9 (H-7) and δ_C_ 43.9 (H-6) were attributable to oxygen-bearing aliphatic tertiary carbons, and C-6 appeared up-field due to a γ-gauche effect (δ_C_ 43.9). Based on the above analysis, six of the seven IHD were consumed by one aromatic ring and two cyclohexane rings, while the remaining IHD could be attributed to an epoxy bridge (between C-6 and C-7), and this was also supported by correlations in the HMBC spectrum (Figure 3). The base peak in the mass spectrum of Compound **1** at *m/z* 284 was [M^+^−16], which also confirmed the existence of an epoxide moiety. The epoxy moiety was further deduced from HMBC correlations of H-6/C-10, H-7/C-5, H-14/C-7, and H-5/C-6 and C-7; COSY correlations between H-6 and H-7; and finally NOESY couplings between H-5 and H_3_-18, H-6 and H_3_-20, H-16 and H-11, and H-14 and H-7. The NOESY spectrum (Figure 4) established a β-*quasi*-equatorial orientation of the two epoxide protons, which was confirmed by their coupling constant *J*(H-6/7) = 4.2 Hz. Additionally, a lower chemical shift at δ_H_ 7.27 for H-14 (1H, s) was due to deshielding by the epoxy group at positions C-6 and C-7. Thus, the structure of Compound **1** was determined to be 6α,7α-epoxyferruginol and given the trivial name Acrocarposporin A.

Compound **2** had a molecular ion peak at *m/z* 314.2250 (HR-EI-MS) and was analyzed for the presence of C_21_H_30_O_2_. The IR spectrum of Compound **2** exhibited the presence of an OH group at 3389 cm^−1^ and aromatic moieties at 1610 and 1490 cm^−1^. The UV absorptions (λ_max_ 232 nm) confirmed the presence of a conjugated C=C bond and an aromatic system. Once again, seven IHD were determined from the molecular formula, the ^13^C-NMR (Table 1), and the DEPT (DEPT-90 and DEPT-135) spectra. Further spectral data (Table 1 and Figure 1) and a comparison with similar compounds [13] established the structure of Compound **2** to be a 20(10→5)-abeo-4,5-seco-abietane naphthalene-type compound. The ^1^H-NMR data indicated the presence of olefinic protons giving rise to AB-type resonances (δ_H_ 7.09 (1H, d, *J* = 8.4 Hz, and H-6) and 7.48 (1H, d, *J* = 8.4 Hz, and H-7)), an iPr group (δ_H_ 3.31 (1H, sep, *J* = 6.8 Hz, and H-15) and 1.33 (6H, d, *J* = 6.8 Hz, and H-16/17)), two aromatic protons on a tetrasubstituted benzene ring (δ_H_ 7.22 (3H, s) and 7.52 (3H; s)), one phenolic OH group (δ_H_ 5.49 (*s*; HO–C(13)), one benzylic Me group at δ_H_ 2.41 (3H, *s*, and Me-5), and a signal characteristic of a 4-methoxy-4-methylpentyl moiety (δ_H_ 2.89 (m; H-1β), 1.23 (m; H-1α), 1.62 (2H, m, and H_2_-2), 1.69 (2H, m, and H_2_-3), 1.15 (6H, s, and CH_3_-18/19), and 3.20 (3H, s, and OMe)). All of these structural features could be readily ascribed to the presence of a 20(10→5)abeo-4,5-secoabieta-5(10),6,8,11,13-pentaen-4,13-diol analog [13].

The ^13^C-NMR and DEPT spectra showed two tetrasubstituted benzene rings, an iPr group, and a 4-methoxy-4-methylpentyl side chain. From the seven IHD, it could be deduced that Compound **2** contained one naphthalene group. With the presence of one benzylic Me group and a side chain (4-methoxy-4-methylpentyl), Compound **2** could be assumed to be a 20(10→5)abeo-4,5-secoabieta-5(10),6,8,11,13-pentaen-4,13-diol compound but with its C-4 bearing a OMe group instead of an OH group. The following HMBC correlations were observed, which supported this assumption: OMe-4 with C-4 (δ_C_ 74.8) and Me-18/19 with C-4 and C-3 (δ_C_ 40.6).

The other HMBC spectrum (Figure 3) revealed the correlations between H3-5/C-6 and 10; H3-16 (17)/C-12, H-11/C-12 and 13; and H-15/C-11, 12, and 13. From the above data, the structure of Compound **2** was unambiguously established as 3-isopropyl-5-(4-methoxy-4-methylpentyl)-6-methylnaphthalen-2-ol and named Acrocarposporin B.

Compound **3**, isolated as an oil, exhibited a molecular ion (M^+^) peak at *m/z* 318.2209 for C_20_H_30_O_3_, corresponding to six indices of hydrogen deficiency (IHD). The IR spectrum of Compound **3** displayed an absorption for an OH group at 3410 cm^−1^ of 1709 (C=O) and bands at 1611 and 1508 (aromatic). Our interpretation of the ^1^H-NMR spectrum of Compound **3** (Table 2) revealed the signals for two methyl groups (δ_H_ 0.71 (3H, s, and Me-19) and 1.08 (3H, s, and Me-18)), an iPr group (δ_H_ 1.15 (3H, d, *J* = 7.0 Hz, and H_3_-16), 1.17 (3H, d, *J* = 7.0 Hz, H_3_-17) and 3.17 (1H, sept, *J* = 7.0 Hz, and H-15)), one OMe attached to an aromatic ring (δ_H_ 3.79 (3H, s, and OMe-13)), and one tetrasubstituted aromatic system with two singlet aromatic protons (δ_H_ 6.51 (1H, s, and H-14); 6.82 (1H, s, and H-11)) in addition to a phenolic hydrogen signal (δ_H_ 7.64, exchangeable) and associated with the carbon to which it was attached (δ_C_ 154.1, C-9), which were all in accordance with the presence of an abietane diterpenoid [14]. However, diterpenoids of the abietane class, in addition to the methyls of the iPr group, should have three singlet methyls; however, Compound **3** had only two present in its ^1^H-NMR spectrum. It can be speculated that one of the singlet methyl groups was absent due to oxidation and decarboxylation. A carbon signal at δ_C_ 216.9 (C-10) was presumed to be a ketone, while from the ^1^H-NMR three protons at δ_H_ 2.35 (m; H1-α), 2.46 (br d, *J* = 15.0 Hz, and H-1β), and 2.21 (m; H-5) were all affected by the ketone group and shifted to a lower magnetic field. Further evidence came from the IHD analysis, which showed that five of the six IHD were consumed by one aromatic ring and one cyclohexane ring, and the remaining IHD were due to the presence of a C=O group. Compound **3** could, therefore, be a 20-norabietane derivative (a 9,10-secoabietane-type compound).

A singlet at δ_H_ 3.79 had a NOESY correlation with H-14 (δ_H_ 6.51) and could, therefore, be assigned as a methoxyl at C-13 (OMe-13). Thus, it was concluded that the remaining signal in the ^1^H spectrum at δ_H_ 6.82 had to have arisen from H-11. The consecutive protons H-5 (δ_H_ 2.21 (1H, m)), CH_2_-6 (δ_H_ 1.62 (2H, m)), and CH_2_-7 (δ_H_ 2.38/2.29 (2H, m)) were revealed from the COSY and NOESY data. The iPr unit was determined to be at C-12 as it had NOESY cross-peaks with δ_H_ 6.82 (1H, s, and H-11). The ^1^H- and ^13^C-NMR chemical shifts and key HMBC, COSY, and NOESY correlations are shown in Table 1 and Table 2 and Figure 3 and Figure 4 and are compatible with the structure of Compound **3** as 20-*nor*-9-hydroxy-12-methoxy-9,10-secoabieta-8,11,13-trien-10-one, which was named Acrocarposporin C.

Compound **4** was obtained as a colorless oil. Its molecular formula was determined as C_21_H_28_O_3_ on the basis of an M^+^ peak at *m/z* 328.2034 (calculated 328.2033 for C_21_H_28_O_3_) in its HREIMS. UV absorptions (λ_max_ 223 and 281 nm) again confirmed the presence of a benzenoid moiety [15]. The IR spectrum of Compound **4** exhibited the presence of an ester at 1745 cm^−1^ and an aromatic moiety at 1633 and 1514 cm^−1^. Eight indices of hydrogen deficiency (IHD) were determined from the molecular formula, ^13^C-NMR (Table 2), and DEPT spectra. The ^1^H-NMR spectrum of Compound **4** indicated the presence of an iPr group (δ_H_ 1.21 (6H, d, *J* = 7.2 Hz, and H-16/17) and δ_H_ 3.19 (1H, sep, *J* = 7.2 Hz, and H-15)), two Me groups (δ_H_ 1.06 (3H, s, and Me-19) and 1.15 (3H, s, and Me-18)) attached to a quaternary C-atom, one OMe (δ_H_ 3.79 (3H, s, and OMe-13)), one methine (δ_H_ 1.78 (1H, d, *J* = 13.0 Hz, and H-5)), two singlet aromatic protons at δ_H_ 6.65 (1H, s, and H-14) and 6.89 (1H, s, and H-11) of a tetrasubstituted benzene, and four aliphatic methylenes. The ^13^C-NMR and DEPT spectra showed signals of an iPr group; two Me groups; one oxygenated methine (δ_C_ 84.2), which was associated with an OC=O group (δ_C_ 177.1); two aromatic C-atoms; and four CH_2_ and two CH groups. The 21 C-signals including two Me and an iPr group were again in accordance with an abietane derivative.

However, as with Compound **3**, abietane diterpenes should normally possess three singlet methyl groups, and Compound **4** only had two in its ^1^H-NMR spectrum. It was speculated that one of the methyl groups may be oxidized to a lactone, which was supported by the presence of an IR absorption band at 1745 cm^−1^ and a ^13^C-NMR signal at δ_C_ 177.1.

A signal in the carbon spectrum at δc 177.1 was presumed to be that of an ester carbonyl group, and, due to HMBC correlations with this carbon between H_2_-1 and H-5, it could be placed at C-20. Further HMBC correlations between an oxymethine signal at δ 4.20 (d, H-3) with C-18, C-19, and C-20 supported the notion that a lactone bridge was present between C-3 and C-10. Therefore, Compound **4** was confirmed as a new lactonic abietane diterpene and was named Acrocarposporin D.

Compound **5** was obtained as a syrup with a specific optical rotation [α]^25^_D_: -71.8 (*c* 0.01; CHCl_3_). The UV spectrum had maximum absorption bands at 220 and 285 nm. The molecular formula was determined as C_22_H_32_O_5_ based on HR-EI-MS data [M]^+^ at *m/z* 376.2263; calc. 376.2260. The IR spectrum pointed to the presence of COOH at 3400-2700 and 1710 cm^−1^, an ester C=O (1745 cm^−1^), and conjugated CO groups (1690 cm^−1^). The ^1^H-NMR spectrum (Table 2) displayed signals of one triple-substituted double bond conjugated with carbonyl groups (δ_H_ 6.60 (1H, t, and *J* = 6.0 Hz)), exomethylene H-atoms (δ_H_ 4.70 (1H; br s) and 4.22 (1H; br s)), and signals of methylene and methyl groups on acetyloxymethylene (CH_2_OAc) (δ_H_ 4.80 (2H, br d, and *J* = 1.2 Hz) and 2.09 (3H, s)). Signals of one C=O C-atom (δ_C_ 200.9 (C-12), 181.4 (C-19), and 171.0 (C-21)), one trisubstituted double bond (δ_C_ 139.6 (C-14) and 133.5 (C-13)), and an exoethylene group (δ_C_ 148.9 (C-8); 106.3 (C-17)) were observed in the ^13^C-NMR and DEPT (DEPT90 and DEPT135) spectra (Table 1), which were responsible for five out of seven unsaturation degrees. The remaining two were ascribed to the presence of two rings. It was inferred to be a bicyclic structure with two six-membered rings; thus, it was deduced to be a labdane diterpenoid.

From the HMBC plot, one oxymethylene at δ_H_ 4.80 (2H, br d, and *J* = 1.2 Hz) is correlated with δ_C_ 170.7, 140.1 (C-14), and 134.6 (C-13), so it was inferred that the signal at δ_H_ 4.80 (2H, br d, and *J* = 1.2 Hz) is between acetoxy and trisubstituted double bonds. Whereas the carbon signal at δ_C_ 200.4 is correlated with H-11 and δ_H_ 6.60 (1H, br d, and *J* = 1.2 Hz); thus, it can be inferred from the above that the ketone group is located at C-12 and δ_H_ 6.60 (1H, br d, and *J* = 1.2 Hz) corresponded to H-13.

According to the NOESY spectrum, H-13/15 were both correlated with H-16 and H-13 was correlated with H-11, which confirms that the double bond is a *Z*-form. The correlation of H-18 with H-5 could be used to determine the equatorial orientation of CH_3_-18. Compound **5** was confirmed to be 12-oxo-15-acetoxylabda-8(17),13(*Z*)-dien-19-oic acid via HSQC and COSY and was named Acrocarposporin E.

## 3. Discussion

Generally speaking, actinomycetes have been developed as a large library that can be expected to provide a variety of structurally unique and pharmacologically active natural products. Secondary metabolites of the genus *Acrocarpospora* have been rarely studied. The *A*. *punica*-type strain 04107M-2^T^ had only 10 components reported by our team in the past [16].

After modifying the fermentation conditions, we obtained five new components from the BuOH active layer and the isolated backbones, including 20(10→5)abeo-4,5-secoabietane naphthalene analogs, abietane, and 20-nor-9,10-secoabietane metabolites. Abietanes are naturally occurring tricyclic diterpenoids that have been isolated from multiple terrestrial plant sources, especially from conifer resins [17,18]. Abietic acid and dehydroabietic acid are the main components of pine resin obtained from *Pinus* sp. [19]. Colophony, the distillation residue of pine oleoresin, is the main source for abietanes, along with conifers other than pines belonging to the Araucariaceae, Cupressaceae, Phyllocladaceae, Pinaceae, and Podocarpaceae families.

Asteraceae, Celastraceae, Hydrocharitaceae, and Lamiaceae, along with some fungal species, are also known to produce abietane diterpenoids [20]. In 2016, a novel norditerpenoid, actinomadurol, was isolated from the rare actinomycete strain, *Actinomadura* sp. KC 191, together with the known compound JBIR-65 [21]. To the best of our knowledge, this is the first report of abietane-type metabolites from the actinomycete genus *Acrocarpospora*. These results demonstrate that *Acrocarpospora* produces unique and diverse metabolites in different fermentation conditions and soil-derived collections. Therefore, in a special ecological environment, more natural products with biological activity may be found by searching for *Acrocarpospora* species.

### Biological Studies

Culture broth from *A*. *punica* 04107M was tested for antifungal activity against the following fungi: *Aspergillus niger* (BCRC-31512), *Penicillium italicum* (BCRC-30567), *Candida albicans* (BCRC-21538), and *Saccharomyces cerevisiae* (BCRC-20822). The antifungal data are shown in Table 3, and the clinically used antifungal drug ketoconazole was employed as a positive control.

Our results indicate that Compounds **1**, **4,** and **5** have moderate antifungal activity compared to ketoconazole, with Compounds **2** and **3** being weaker. From the results of the antifungal tests, the following conclusions can be drawn about these isolates: (a) within the novel strain, the 6α,7α-epoxyferruginol (Compound **1**) and 20(10→5)abeo-4,5-secoAbietane diterpene naphthalene ring-type compound (Compound **2**) showed antifungal activities with inhibition zones of 27, 18, 27, and 29 mm; there were inhibition zones of 22, 18, 17, and 28 mm against *Aspergillus niger* (BCRC-31512), *Penicillium italicum* (BCRC-30567), *Candida albicans* (BCRC-21538), and *Saccharomyces cerevisiae* (BCRC-20822), respectively. (b) The 20-norabietane-type (9,10-secoabietane-type) Acrocarposporin C (Compound **3**) exhibited weak antifungal activities against the *Aspergillus niger* (BCRC-31512), *Penicillium italicum* (BCRC-30567), *Candida albicans* (BCRC-21538), and *Saccharomyces cerevisiae* (BCRC-20822) strains. (c) The other type of *O*-Methylpisiferic acid, Acrocarposporin D (Compound **4**), indicated effective inhibition zones of 22, 26, 29, and 28 mm against *Aspergillus niger* (BCRC-31512), *Penicillium italicum* (BCRC-30567), *Candida albicans* (BCRC-21538), and *Saccharomyces cerevisiae* (BCRC-20822), respectively. (d) The labdane-type structure in the diterpenoids Compound **5** and Acrocarposporin E (Compound **3**) exhibited moderate antifungal activities against the *Aspergillus niger* (BCRC-31512) and *Penicillium italicum* (BCRC-30567) strains (Table 3).

The inhibitory activity of Compounds **1**, **2**, **4**, and **5** against *A*. *niger*, *P*. *italicum*, *C*. *albicans*, and *S*. *cerevisiae* was further tested using the method described in the experimental section (Table 4). Compound **1** has inhibitory activity against *A*. *niger*, *P*. *italicum*, and *C*. *albicans* strains with MIC values of 54.87, 53.98, and 49.56 μg/mL. Compound **2** has inhibitory activity against *Saccharomyces cerevisiae* with an MIC value of 57.38 μg/mL. Compound **4** was found to have moderate inhibitory activity against the *P*. *italicum*, *C*. *albicans*, and *S*. *cerevisiae* strains with MIC values ranging from 38.89 to 42.78 μg/mL. Compound **5** also had MIC values of 59.78, 51.32, and 56.92 μg/mL against *A*. *niger*, *C*. *albicans*, and *S*. *cerevisiae*, respectively. They were less biologically active than the reference compound, ketoconazole, which had MIC values of 3.25, 6.72, 11.79, and 3.16 μg/mL against *A*. *niger*, *Pseudomonas italia*, *C*. *albicans*, and *S*. *cerevisiae*, respectively. In this bioassay, no antifungal activity (MIC > 100) was observed for Compound **3** at concentrations below 100 μg/mL.

## 4. Materials and Methods

### 4.1. General Experimental Procedures

For the TLC, we used silica gel 60 F254-precoated plates (Merck); for column chromatography (CC), we used silica gel 60 (70–230 or 230–400 mesh, Merck) and Spherical C18 100A Reversed Phase Silica Gel (RP-18) (particle size: 20–40 μm) (Silicycle). For the HPLC analysis, we used a spherical C18 column (250 × 10 mm, 5 μm) (Waters) and LDC-Analytical-III apparatus. For the UV spectra, we used a Jasco UV-240 spectrophotometer, with λmax (log ε) in nm. For optical rotation, we used a Jasco DIP-370 polarimeter, in CHCl3. For the IR spectra, we used a Perkin-Elmer-2000 FT-IR spectrophotometer, with ν in cm^−1^. For the ^1^H-, ^13^C-, and 2D-NMR spectra, we used Varian-VNMRS-600 and Varian-Unity-Plus-400 spectrometers; δ in ppm relative to Me4Si, *J* in Hz. For the ESI and HRESIMS, we used a Bruker APEX-II mass spectrometer, in *m/z*.

### 4.2. Microorganism, Cultivation, and Preparation of the Actinobacteria Strain

This strain was isolated from soil samples collected in Taitung County, Taiwan, using HV agar and cultured at 28 °C for 3 weeks. The strain was kept on oat agar and the spores or mycelial fragments of the strain were suspended in broth containing 20% (v/v) glycerol and stored at –20 °C. The medium for the inoculum contained malt extract, 3 g; yeast extract, 3 g; glucose, 5 g; agar, 1.5 g; and 1 L of distilled water. The initial pH of the medium was 8. The synthetic medium contained Glucose, 20 g; Sodium Glutamate (MSG), 10 g; K2HPO4, 5 g; KH2PO4, 5 g; MgSO4•7H2O, 1.0 g; KCl, 0.5 g; ZnSO4•7H2O, 0.01 g; FeSO4•7H2O, 0.01g; and MnSO4•H2O, 0.003 per liter of distilled water. The initial pH of the medium was adjusted to 5.5. The tilted cultures were maintained on Difco potato dextrose agar (PDA). Spores of the strain were prepared by growing them on PDA slants at 28 °C for 14 days. The spores were washed with sterile water. A 5L Erlenmeyer flask was incubated containing 2L of inoculation medium with the suspension of 107 spores for 3 days at 28 °C on a rotary shaker. This inoculum was transferred to a 100 L fermenter (B. Braun, Germany) containing 30 L of synthetic medium, which was operated at 100 rpm and 30 °C with an aeration rate of 0.3 vvm. After 14 days of culture, fermentation was stopped, and the liquid culture was separated from the mycelium by filtration.

### 4.3. Isolation and Characterization of Secondary Metabolites

The culture filtrate (20 l) was repeatedly extracted with BuOH five times. The pooled BuOH fraction was evaporated under vacuum to yield 78 gm of dark brown residue; the extract was then applied to normal-phase column chromatography packed with silica gel and eluted with methylene chloride/ethyl acetate/acetone/methanol in a stepwise gradient mode to yield 9 fractions (1 to 9). Fraction 3 (2897 mg) was purified by normal-phase MPLC, which was eluted with CH2Cl2-EtOAc (5:1) to afford Acrocarposporin B (Compound **2**) (4.2 mg).

Fraction 4 (12.1 g) was subjected to normal-phase MPLC and eluted with MeOH/H2O (1:1) to yield 5 fractions (4-1 to 4-5). Fraction 4-5 (588 mg) was subjected to Sephadex LH-20 and eluted with MeOH to yield Acrocarposporin C (Compound **3**) (3.8 mg). Fraction 5 (3.98 g) was subjected to silica gel, eluted with CH2Cl2, and then enriched with acetone to yield 10 fractions (5-1 to 5-10). Fraction 5-3 (723 mg) was chromatographed on a CC (20 g, SiO2, and 230–400 mesh; n-hexane/acetone 2:1) to afford Acrocarposporin E (Compound **5**) (3.6 mg). Fraction 5-8 (878 mg) was subjected to silica gel chromatography and eluted with CH2Cl2-MeOH step gradients to yield Acrocarposporin D (Compound **4**) (6.72 mg) and Acrocarposporin A (Compound **1**) (8.1 mg).

Acrocarposporin A (Compound **1**): colorless oil; [α${]}_{D}^{24}$ = −88.2 (*c* 0.01, CHCl_3_); UV (MeOH): 234 (4.11), 280 (3.95) nm; IR (KBr): 3340 (OH), 1610 (aromatic ring), 1510, 1233 (epoxide), cm^−1^; ^1^H NMR (600 MHz, CDCl_3_): see Table 2; ^13^C NMR (150 MHz, CDCl_3_): see Table 1); EIMS (70 eV) *m/z* (%):300 ([M]^+^, 22), 284 ([M]^+^–16, 100), 269 (19), 244 (15), 212 (35), and 202 (40); HRESIMS *m/z* 300.2092 [M]^+^ (calculated for C_20_H_28_O_2_, 300.2094).

Acrocarposporin B (Compound **2**): oil; UV (MeOH): 232 (4.28) nm; IR (Neat): 3389 (OH), 1610, 1490 (aromatic ring) cm^−1^; ^1^H NMR (600 MHz, CDCl_3_): see Table 2; ^13^C NMR (150 MHz, CDCl_3_): see Table 1); EIMS (70 eV) *m/z* (%): 314 ([M]^+^, 29), 282 (13), 239 (5), 226 (3), 213 (100), 207 (3); HRESIMS *m/z* 314.2248 [M]^+^ (calculated for C_21_H_30_O_2_, 314.2248).

Acrocarposporin C (Compound **3**): oil; UV (MeOH): 242 (4.09), 285 (3.90) nm; IR (Neat): 3410 (OH), 1709 (C=O), 1611, 1508 (aromatic ring) cm^−1^; ^1^H-NMR (600 MHz, CDCl_3_): see Table 2; ^13^C-NMR (150 MHz, CDCl_3_): see Table 1; EIMS (70 eV) *m/z* (%): 318 ([M]^+^, 28), 300 (16), 285 (32), 192 (100), 179 (98), 165 (35); HRESIMS *m/z* 318.2209 [M]^+^ (calculated for C_20_H_30_O_3_, 318.2207).

Acrocarposporin D (Compound **4**): oil; [α${]}_{D}^{25}$ = +19.8 (*c* 0.01, CHCl_3_); UV (MeOH): 223 (4.33), 281 (3.98) nm; IR (Neat): 1745 (OC=O), 1633, 1514 (aromatic ring) cm^−1^; ^1^HNMR (600 MHz, CDCl_3_): see Table 2; ^13^CNMR (150 MHz, CDCl_3_): see Table 1; EIMS (70 eV) *m/z* (%): 328 ([M]^+^, 22), 307 (15), 289 (12), 154 (100); HRESIMS *m/z* 328.2034 [M]^+^ (calculated for C_21_H_28_O_3_, 328.2033).

Acrocarposporin E (Compound **5**): oil; [α${]}_{D}^{25}$ = −71.8 (*c* 0.01, CHCl_3_); UV (MeOH): 220 (3.18), 285 (4.71) nm; IR (Neat): 1710 (COOH), 1745 (ester), 1690 (conjugated C=O) cm^−1^; ^1^H-NMR (600 MHz, CDCl_3_): see Table 2; ^13^C-NMR (150 MHz, CDCl_3_): see Table 1; EIMS (70 eV) *m/z* (%): 376 ([M]^+^, 5), 353 (4), 283 (20), 213 (20), 199 (10), 165 (13), 136 (70), 107 (31); HRESIMS *m/z* 376.2263 [M]^+^ (calculated for C_22_H_32_O_5_, 376.2260).

### 4.4. Antifungal Activity Assays

The assays tested for the presence of microorganisms. The in vitro antifungal activity of Compounds **1**–**5** was tested against a panel of laboratory control strains belonging to the Bioresource Collection and Research Center (BCRC) in Hsinchu, Taiwan, namely, the fungal organisms *Aspergillus niger* (BCRC-31512), *Penicillium italicum* (BCRC-30567), *Candida albicans* (BCRC-21538), and *Saccharomyces cerevisiae* (BCRC-20822).

#### 4.4.1. By Disk Diffusion Assay

Antifungal susceptibility testing of the isolated compounds was performed with the following strains: *Aspergillus niger*, *Penicillium italicum*, *Candida albicans*, and *Saccharomyces cerevisiae* by the disk diffusion method and the following CLSI guidelines were applied: M44-A and M44-S2 for yeasts [22,23] and M-51P for filamentous fungi [24]. A standard disk of ketoconazole was used as a positive control, while a disk imbued with 50 μL of pure DMSO was used as a negative control. The diameters of the inhibition zones were measured in millimeters by means of a slide caliper. Each test was performed in triplicate, and the results were analyzed for statistical significance [22,23,24].

#### 4.4.2. By Broth Dilution Assay

The MIC determination for the antifungal assay was performed according to the Clinical and Laboratory Standard Institute (CLSI) using the broth dilution assay method [25,26,27]. Extract stock solutions and partitions were prepared in 5% DMSO, and twofold serial dilutions were prepared in RPMI in 96-well microtiter plates (Corning Incorporated, Corning, NY, USA). The final concentrations ranged from 0.98 to 2.000 g mL^−1^. Test organisms (100 μL) were added to each well in microtiter plates. The growth control contained medium and inoculum. Blank controls contained medium only. The microtiter plates were then incubated at 35 °C and the endpoints were read after 48 h. The lowest concentration for each test compound at which color change occurred was recorded as its primary MIC value. The average of primary values from three individual tests were calculated, and the average was taken as the final MIC value for each of the test compounds.

## 5. Conclusions

Actinomycetes have potential economic and biotechnological value and have long been recognized as major microorganisms in the medical industry. To date, there are tens of thousands of antibiotics produced by microorganisms, of which more than 70% are derived from *Actinobacteria* [28]. The secondary metabolites of *Actinobacteria* have various structures and biological activities, including antibacterial, antifungal, antitumor, insecticidal, and herbicidal properties; enzyme inhibitory activity; and immune regulatory activity [29,30], indicating that *Actinobacteria* have great potential with respect to the development of new medicine. As part of our investigations aimed at exploring structurally novel, bioactive secondary metabolites from actinomycetes, the chemical research on the fermentation extract of *Acrocarpospora punica* led to the isolation of five previously undescribed compounds, namely, Acrocarposporins A–E (Compounds **1**–**5**) (Figure 2). The structures of these isolates were determined by spectroscopic experiments. The BuOH soluble fraction from the *A*. *punica* fermentation broth was tested for antifungal activities. Our results indicated that Compounds **1**, **2**, **4**, and **5** displayed moderate antifungal activities against *Aspergillus niger*, *Penicillium italicum*, *Candida albicans*, and *Saccharomyces cerevisiae*. It is worth mentioning that the chemical composition of *Acrocarpospora punica* 04107M has never been studied. This is the first report on diterpenoid metabolites from the genus *Acrocarpospora*.

## Figures and Tables

**Table 1:** ^13^C-NMR data for Compounds **1**–**5** (*δ* in ppm, 150 MHz for ^13^C NMR in CDCl_3_).

No.	1	2	3	4	5
1	39.6	29.3	41.8	32.6	39.2
2	18.8	24.2	23.0	21.7	19.8
3	45.5	40.6	40.2	84.2	37.9
4	34.8	74.8	40.0	38.0	44.1
5	47.9	133.7	60.0	50.4	55.9
6	43.9	126.8	24.8	22.9	25.6
7	76.9	125.5	30.2	30.0	37.9
8	126.9	128.3	118.3	128.5	148.9
9	149.1	131.7	154.1	132.5	50.8
10	38.8	132.3	216.9	46.1	39.7
11	109.1	106.0	99.7	110.8	33.1
12	153.5	152.3	156.4	155.1	200.9
13	132.8	135.2	127.9	136.2	134.6
14	128.1	125.8	126.6	126.4	140.1
15	26.7	27.4	26.2	26.6	61.4
16	22.9	22.6	22.9	22.5	12.1
17	22.3	22.6	22.9	22.7	106.3
18	36.5	24.9	29.9	27.6	28.9
19	23.2	24.9	21.0	21.7	181.4
20	26.2	20.1	55.5	177.1	13.4
21		49.1		55.8	171.0
22					29.0

**Table 2:** ^1^H-NMR data for Compounds **1**–**5** in CDCl_3_ (*δ* in ppm, *J* in Hz, and 600 MHz in CDCl_3_).

No.	1	2	3	4	5
1	1.50 (m, H-α) 2.28 (br d, *J* = 13.2 Hz, H-β)	1.23 (m, H-α) 2.89 (m, H-β)	2.46(br d, *J* = 15.0 Hz, H-β) 2.35 (m, H-α)	1.84 (1H, m, H-α) 2.32 (1H, td, *J* = 13.0, 5.0 Hz, H1-β)	1.18/1.61 (each 1H, m)
2	1.71 (1H, m) 1.59 (1H, m)	1.62 (2H, m)	1.82 (1H, m) 1.96 (1H, m)	2.15 (2H, m)	1.50/1.85 (each 1H, m)
3	1.39 (2H, m)	1.69 (2H, m)	1.59 (1H, m) 1.74 (1H, m)	4.20 (1H, d, *J* = 4.0 Hz, H-β)	1.05/2.20 (each 1H, m)
4					
5	1.97 (1H, d, *J* = 10.8 Hz)		2.21 (1H, m)	1.78 (d, *J* = 13.0)	1.47 (1H, dd, *J* = 12.8, 2.6 Hz)
6	3.40 (1H, dd, *J* = 10.8, 4.2 Hz)	7.09 (d, *J* = 8.4)	1.62 (2H, m)	1.38 (1H, m) 1.86 (1H, m)	1.85–1.98 (m)
7	5.18 (1H, br d, *J* = 4.2 Hz)	7.48 (d, *J* = 8.4)	2.38 (1H, m) 2.29 (1H, m)	2.76 (1H, m) 2.67 (1H, m)	2.15–2.40 (m)
8					
9					2.60 (m)
10					
11	6.68 (s)	7.22 (s)	6.82 (s)	6.89 (1H, s)	2.59–2.63 (m, H-11β) 2.96 (dd, *J* = 16.5, 10.5 Hz, H-11α)
12					
13					6.60 (br t, *J* = 1.2 Hz))
14	7.25 (s)	7.52 (s)	6.51 (s)	6.65 (1H, s)	
15	3.06 (1H, sep, *J* = 7.4 Hz)	3.31 (1H, sep, *J* = 6.8 Hz)	3.17 (1H, sep, *J* = 7.0 Hz)	3.19 (1H, sep, *J* = 7.2 Hz)	4.80 (br d, *J* = 1.2 Hz)
16	1.23 (3H, d, *J* = 7.4 Hz)	1.33 (3H, d, *J* = 6.8 Hz)	1.15 (3H, d, *J* = 7.0 Hz)	1.21 (3H, d, *J* = 7.2 Hz)	1.80 (br s)
17	1.23 (3H, d, *J* = 7.4 Hz)	1.33 (3H, d, *J* = 6.8 Hz)	1.17 (3H, d, *J* = 7.0 Hz)	1.21 (3H, d, *J* = 7.0 Hz)	4.70/4.22 (each 1H, br s)
18	1.26 (3H, s)	1.15 (3H, s)	1.08 (3H, s)	1.15 (3H, s)	1.24 (s)
19	1.29 (3H, s)	1.15 (3H, s)	0.71 (3H, s)	1.06 (3H, s)	
20	1.22 (3H, s)	2.41 (3H, s)	3.79 (3H, s)		0.67 (3H, s)
21		3.20 (3H, s)		3.79 (3H, s)	
22					2.09 (3H, s)

**Table 3:** Antifungal activity of six sufficient compounds isolated from the culture broth of *A*. *punica* 04107M (diameter of the zone of growth-inhibitory fungicidal zone is given in mm, including the diameter of the disk, which is 8 mm).

Test Microorganism	Isolated Compounds					
	1	2	3	4	5	Ketoconazole
*A*. *niger*	27.4 ±1.7	22.7 ± 1.2	22.3 ± 1.9	22.0 ± 2.8	27.5 ± 2.8	36 ± 1.7
*P*. *italicum*	18.7 ± 0.3	18.2 ± 2.6	19.4 ± 1.2	26.3 ± 1.8	19.3 ± 0.3	33 ± 1.8
*C*. *albicans*	27.2 ± 1.4	17.0 ± 1.2	16.2 ± 2.6	29 ± 2.3	28.0 ± 1.3	39 ± 4.4
*S*. *cerevisiae*	29.2 ± 2.4	28.1 ± 1.5	11.2 ± 2.7	28 ± 2.3	27.3 ± 1.4	34 ± 1.9

Inhibitory zone diameter (mm); + inhibitory zone; positive control (STD): ketoconazole. Each value represents the mean ± SD.

**Table 4:** MIC values of Compounds **1**–**5** in μg/mL against four fungi strains.

Compounds	*A*. *niger*	*P*. *italicum*	*C*. *albicans*	*S*. *cerevisiae*
**1**	54.87 ± 6.13 ^a^	53.98 ± 2.34 ^a^	49.56 ± 6.49 ^a^	>100
**2**	>100	>100	>100	57.38 ± 7.28 ^a^
**3**	>100	>100	>100	>100
**4**	>100	42.78 ± 5.23 ^a^	38.89 ± 3.31 ^a^	40.58 ± 0.23 ^a^
**5**	59.78 ± 7.04 ^a^	>100	51.32 ± 10.60 ^a^	56.92 ± 13.19 ^a^
Ketoconazole	3.25 ± 1.48 ^a^	6.72 ± 2.23 ^a^	11.79 ± 4.81 ^a^	3.16 ± 1.51 ^a^

^a^ Each value represents the mean ± SD.

## Data Availability

Not applicable.

## Supplementary Materials

The supplementary materials can be downloaded at: https://www.mdpi.com/article/10.3390/antibiotics12010095/s1.
