# Supplementary material for: Antifungal Activities of Compounds Produced by Newly Isolated Acrocarpospora Strains
Source: Antibiotics (Basel). 2023 Jan 5;12(1):95. doi: 10.3390/antibiotics12010095 (PMC9854854; doi:10.3390/antibiotics12010095)
Supplement: Supplementary file 1 [file antibiotics-12-00095-s001.zip › antibiotics-2022910-supplementary.pdf]

Supplementary File

Antifungal Activities of Compounds Produced by Newly Isolated *Acrocarpospora* Strains

Ming-Jen Cheng[1*], Jih-Jung Chen[2,3], Ming-Der Wu[4], Jyh-Yih Leu[1], and Min Tseng[4]

[1] Department of Life Science, Fu Jen Catholic University, New Taipei City, 24205, Taiwan
[2] Department of Pharmacy, School of Pharmaceutical Sciences, National Yang Ming Chiao Tung University (NYCU), Taipei 112, Taiwan
[3] Department of Medical Research, China Medical University Hospital, China Medical University, Taichung 404, Taiwan
[4] Bioresource Collection and Research Center (BCRC), Food Industry Research and Development Institute (FIRDI), Hsinchu 300, Taiwan
* Correspondence: chengfirdi@gmail.com (M.-J.C.)

Contents

Table S1. Physiological characteristics of strain 04107M 4

Table S2. DNA hybridization rates among species of *Acrocarpospora* 5

Figure S1. ^{1}H NMR spectrum of **1** 6

Figure S2. ^{13}C NMR spectrum of **1** 6

Figure S3. ^{1}H-^{1}H COSY spectrum of **1** 7

Figure S4. HMBC spectrum of **1** 7

Figure S5. NOESY spectrum of **1** 8

Figure S6. HSQC spectrum of **1** 8

Figure S7. ^{1}H NMR spectrum of **2** 9

Figure S8. ^{13}C NMR spectrum of **2** 9

Figure S9. ^{1}H-^{1}H COSY spectrum of **2** 10

Figure S10. HMBC spectrum of **2** 10

Figure S11. NOESY spectrum of **2** 11

Figure S12. HSQC spectrum of **2** 11

Figure S13. ^{1}H NMR spectrum of **3** 12

Figure S14. ^{13}C NMR spectrum of **3** 12

Figure S15. ^{1}H-^{1}H COSY spectrum of **3** 13

Figure S16. HMBC spectrum of **3** 13

Figure S17. NOESY spectrum of **3** 14

Figure S18. HSQC spectrum of **3** 14

Figure S19. ^{1}H NMR spectrum of **4** 15

Figure S20. ^{13}C NMR spectrum of **4** 15

Figure S21. ^{1}H-^{1}H COSY spectrum of **4** 16

Figure S22. HMBC spectrum of **4** 16

Figure S23. NOESY spectrum of **4**..17
Figure S24. HSQC spectrum of **4** 17

Taxonomic Identification (Phenotypic and Genotypic Data) of Acrocarpospora punica 04107M

Strain 04107M produced branched and non-fragmented substrate mycelia, with club-shaped structures borne on the tips of the aerial mycelium. The spores were non-motile, rod-like, and smooth-surfaced (Figure 1). Their growths on various media were poor; a reddish color with golden lustrous crystals was observed on the oatmeal agar. No soluble pigment was produced in all of the media tested. The strain's physiological and biochemical test results are shown in Table 1.

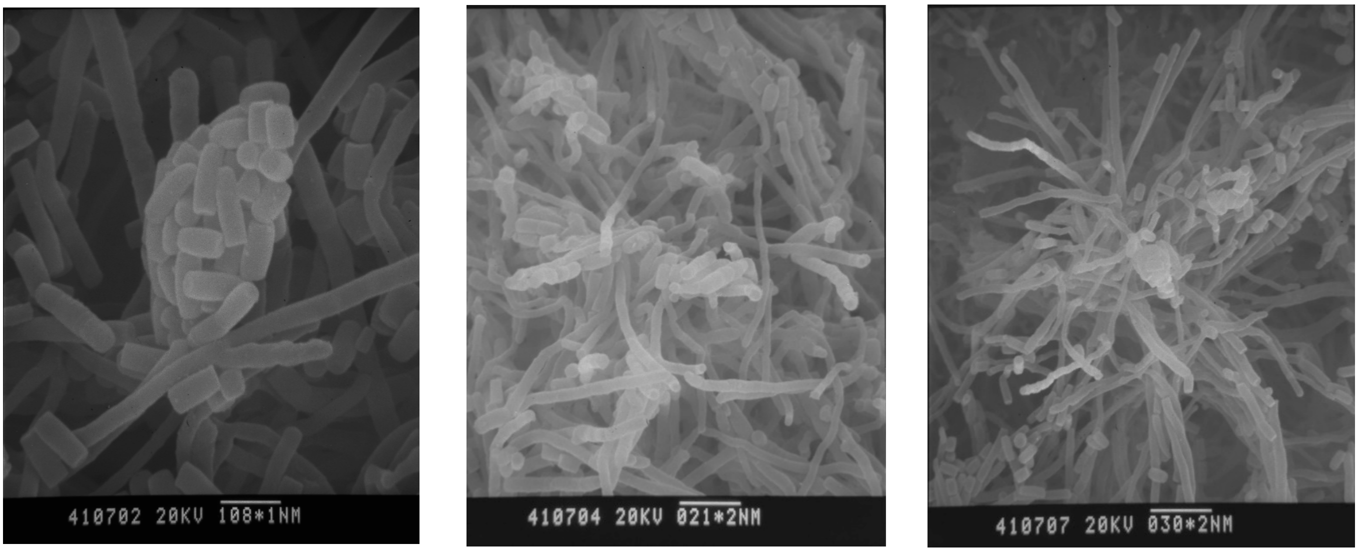

Scanning electron micrographs of strain *A. punica* 04107M grown on HV agar for 14 days at 28 °C. Bar: 1.5 μm.

Cellular biochemistry

Strain 04107M contained meso-A_2pm, madurose, arabinose, rhamnose, glucose, and ribose in whole-cell hydrolysates. The predominant menaquinones found were MK-9(H_4) and MK-9(H_2); mycolic acids were not detected. Phosphatidylethanolamine (PE) was detected. The major fatty acid methyl esters were Iso-$C_{16:0}$ (14.82%), $C_{16:0}$ (14.63%), $C_{17:0}$ (13.79%), and 10-methyl$C_{17:0}$ (23.77%). The G + C content of the DNA was 72.2 mol%.

Phylogeny

The nearly complete 16S rRNA gene sequence (1511 nt) of strain 04107M^T was determined. A preliminary comparison of the sequence against the GenBank database revealed high sequence similarity values with members of the genus *Acrocarpospora*. The phylogenetic tree based on the 16S rRNA gene sequences of the strain 04107M^T and the other valid published *Acrocarpospora* species and other related species is shown in Figure 2. The binary similarity values ranged between 96.5% (*A. pleiomorph* IFO 16266^T) and 98.2% (*A. corrugata* NBRC 13972^T). The DNA–DNA hybridization rates determined for the new isolate 04107M^T to its closest strain types of *A. corrugata* BCRC 16357^T, *A. macrocephala*, and *A. pleiomorpha* were 2.2%, 0.5%, and 7.0%, respectively (Table 2). The distinctiveness of the isolate also stems from phenotypic evidence compared with the nearest phylogenetic neighbors (Table 2). On the basis of phenotypic and genotypic characters, it was evident that the isolate should be classified as a new species of the genus *Acrocarpospora*. Its name is *Acrocarpospora punica* sp. nov., with the strain type 04107M^T.

Table S1. Physiological characteristics of strain 04107M.

**Characteristics**	**Reaction**
Growth temperature (°C)	22–35
Decomposition of:	
Adenine	-
Aesculin	+
Casein	+
Hypoxanthine	-
L-tyrosine	-
Xanthine	-
Production of:	
Amylase	-
Melanin	-
Nitrate reductase	-
Urease	+

+: positive reaction; -: negative reaction.

Table S2. DNA hybridization rates among species of *Acrocarpospora*.

**Probe**	**04107M^{T} (%)**	***A. corrugata*^{T} (%)**
04107M^{T}	100	14.9
A. corrugata^{T}	2.2	100
A. macrocephala^{T}	0.5	4.5
A. pleiomorpha^{T}	7	8.6

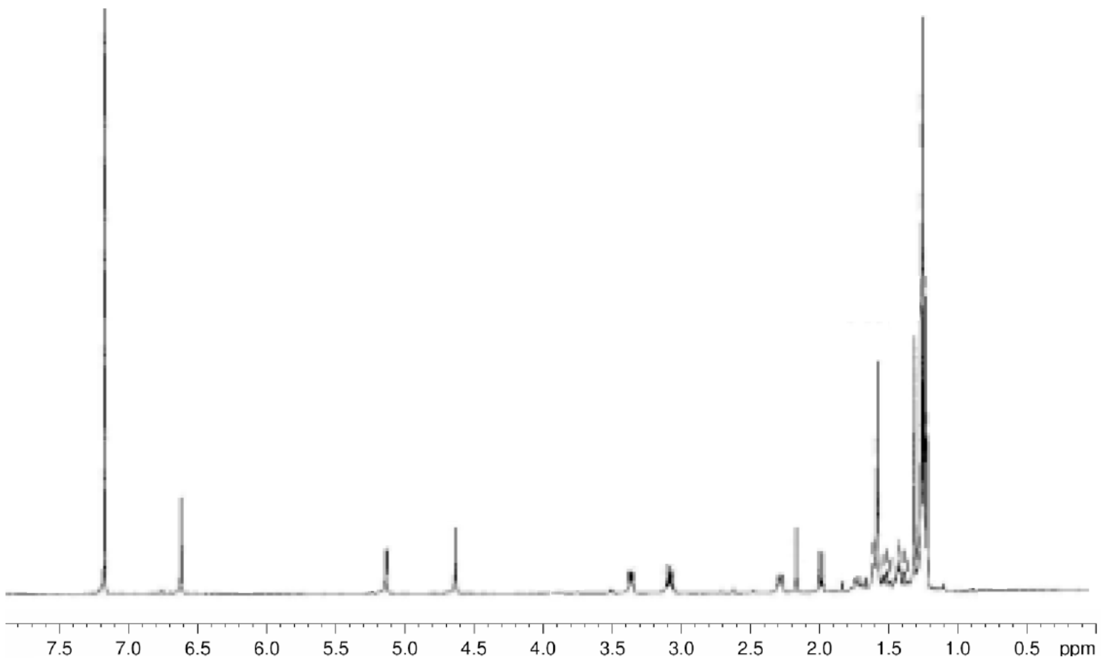

Figure S1. ^{1}H NMR spectrum of **1**

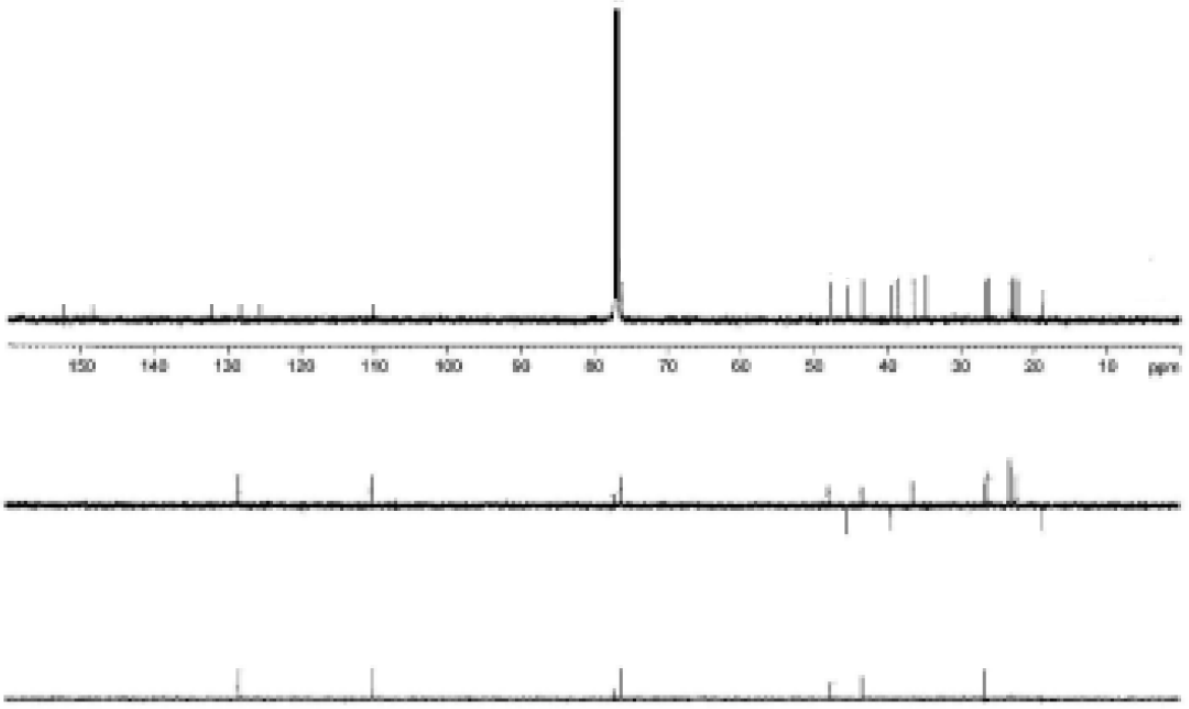

Figure S2. ^{13}C NMR & DEPT spectra of **1**

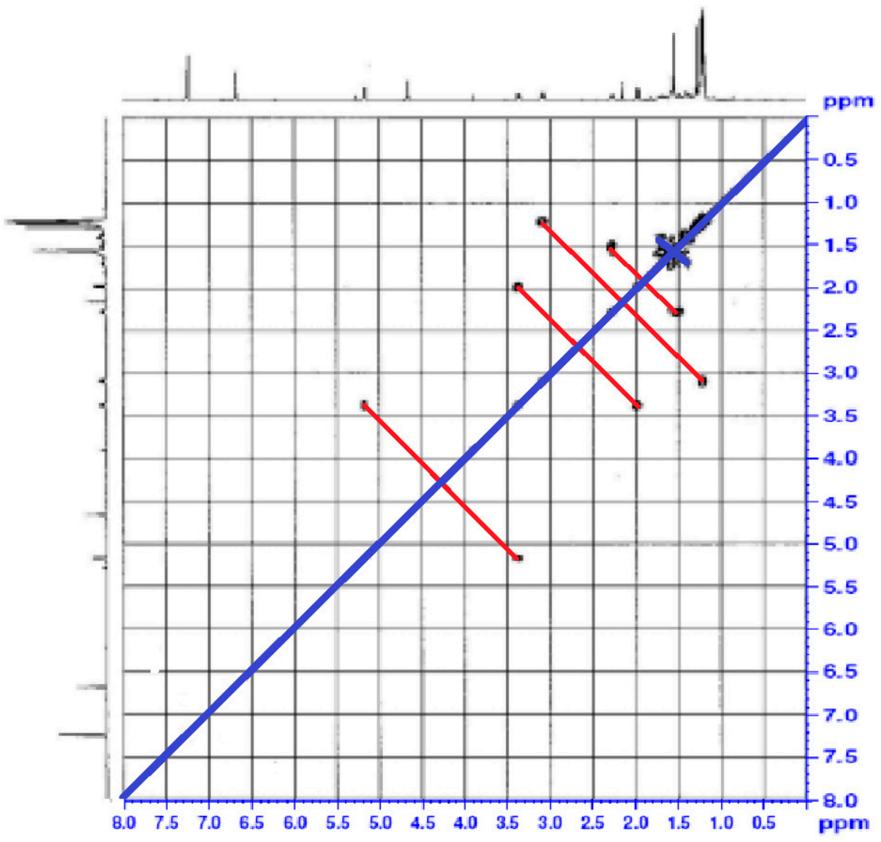

Figure S3. COSY spectrum of **1**

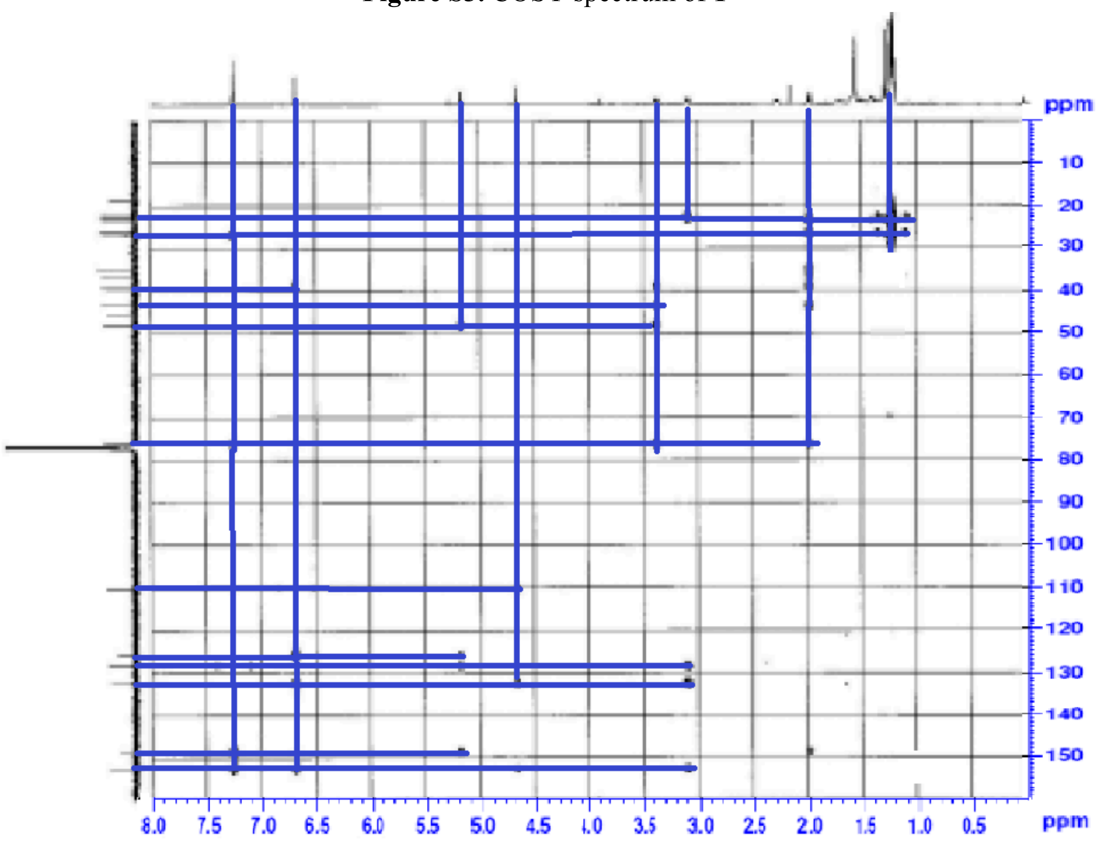

Figure S4. HMBC spectrum of **1**

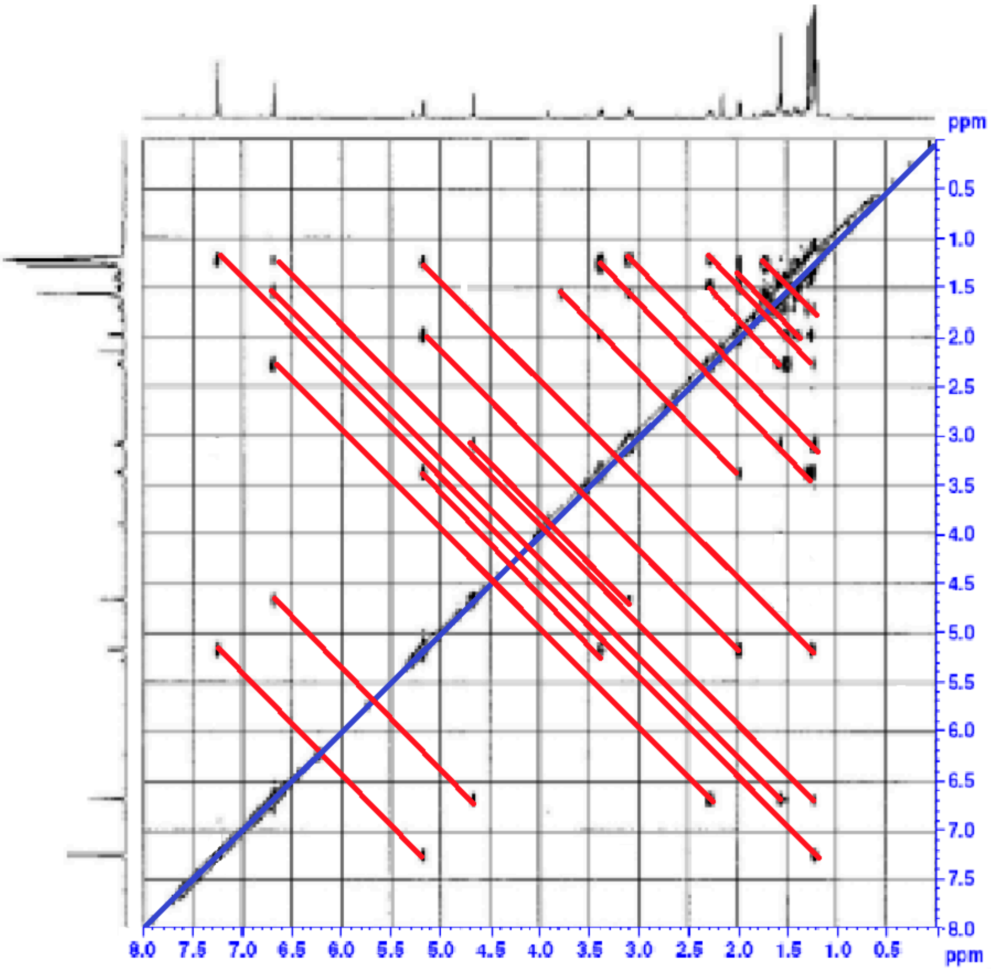

Figure S5. NOESY spectrum of **1**

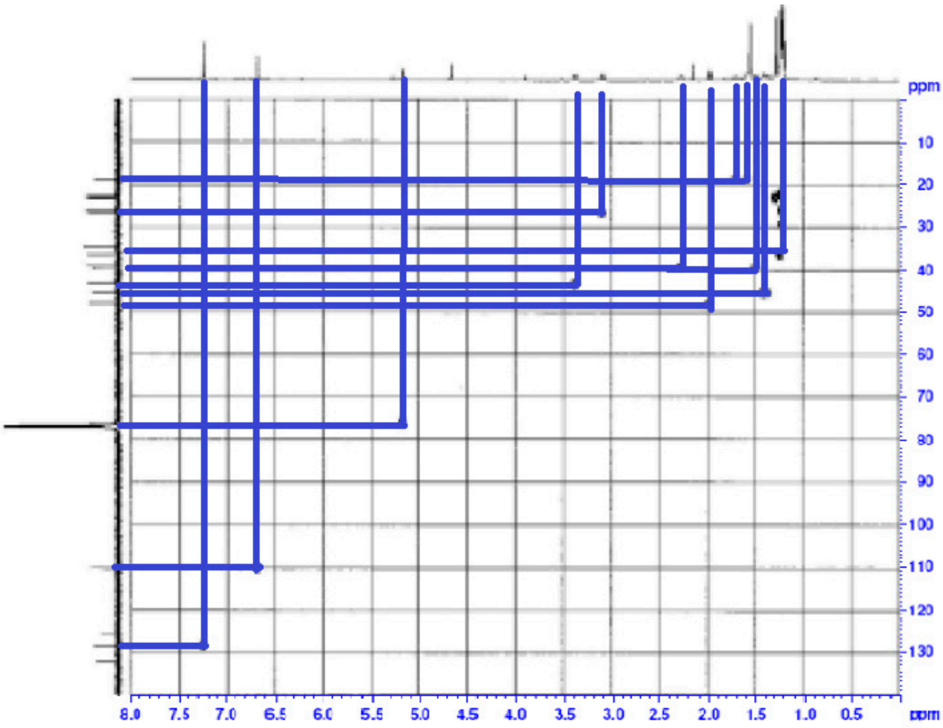

Figure S6. HSQC spectrum of **1**

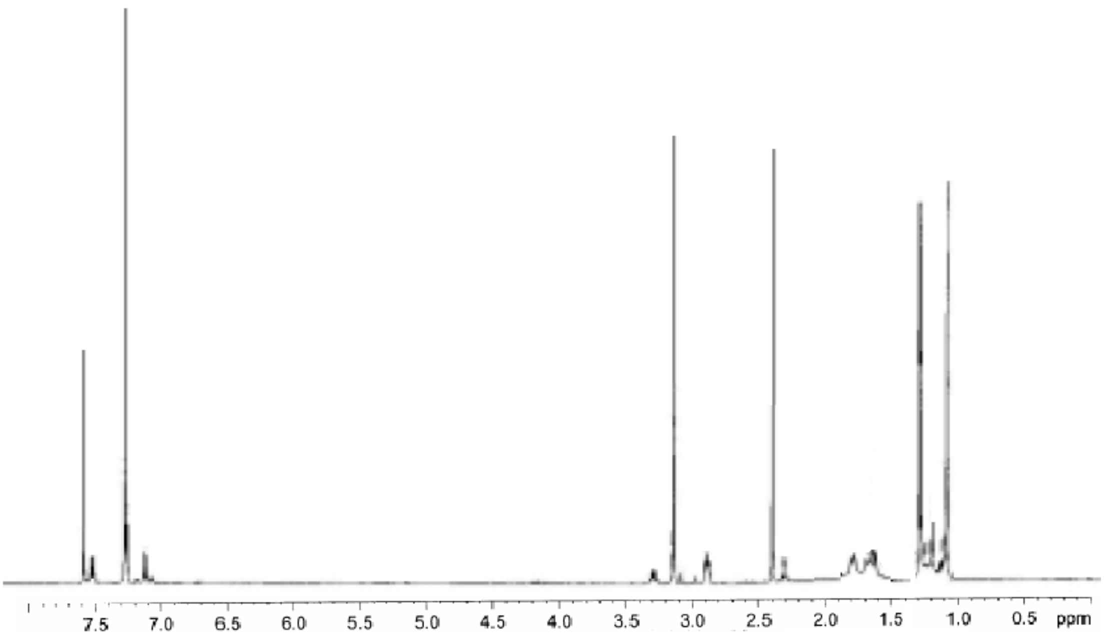

Figure S7. ^{1}H NMR spectrum of **2**

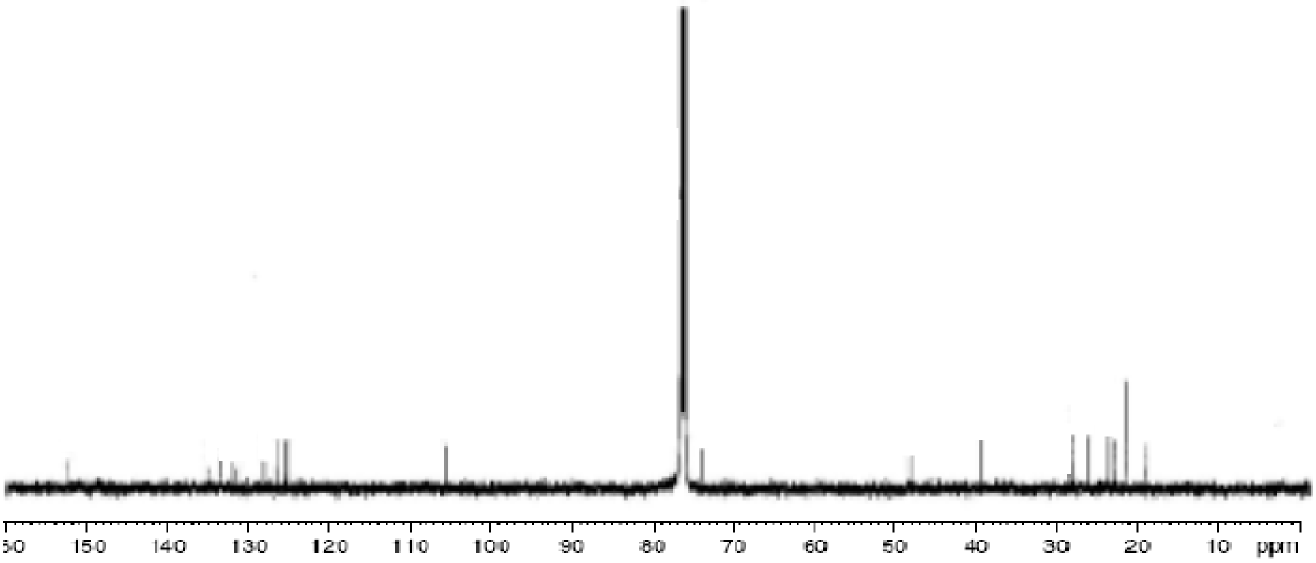

Figure S8. ^{13}C spectrum of **2**

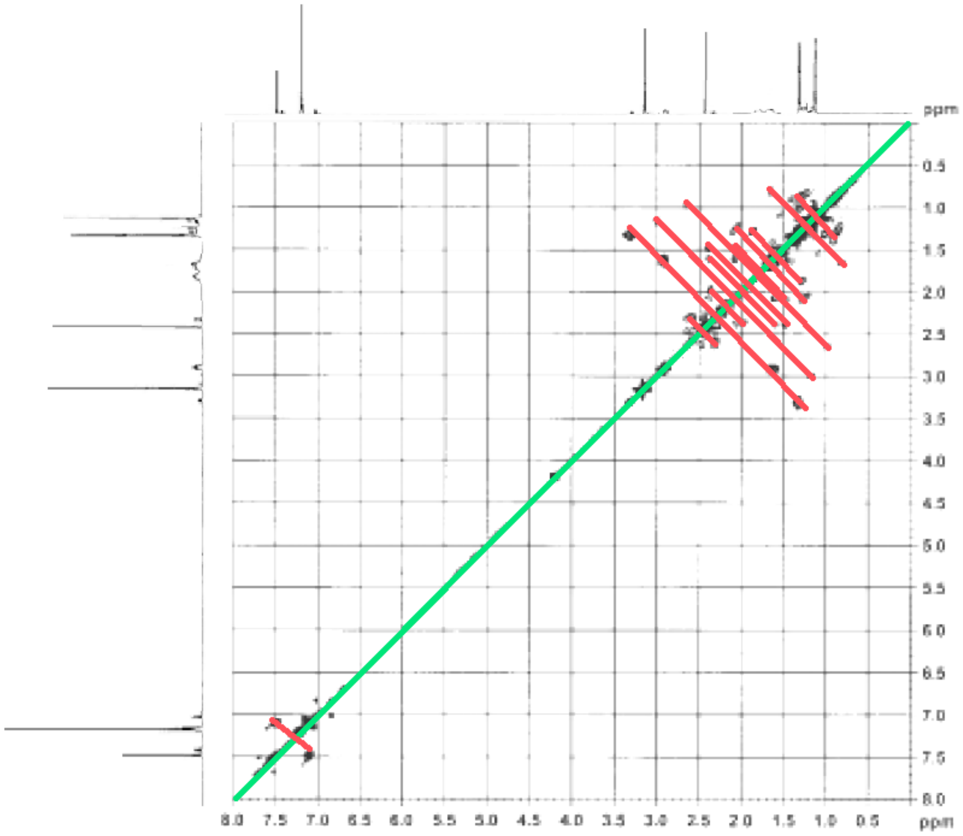

Figure S9. COSY spectrum of **2**

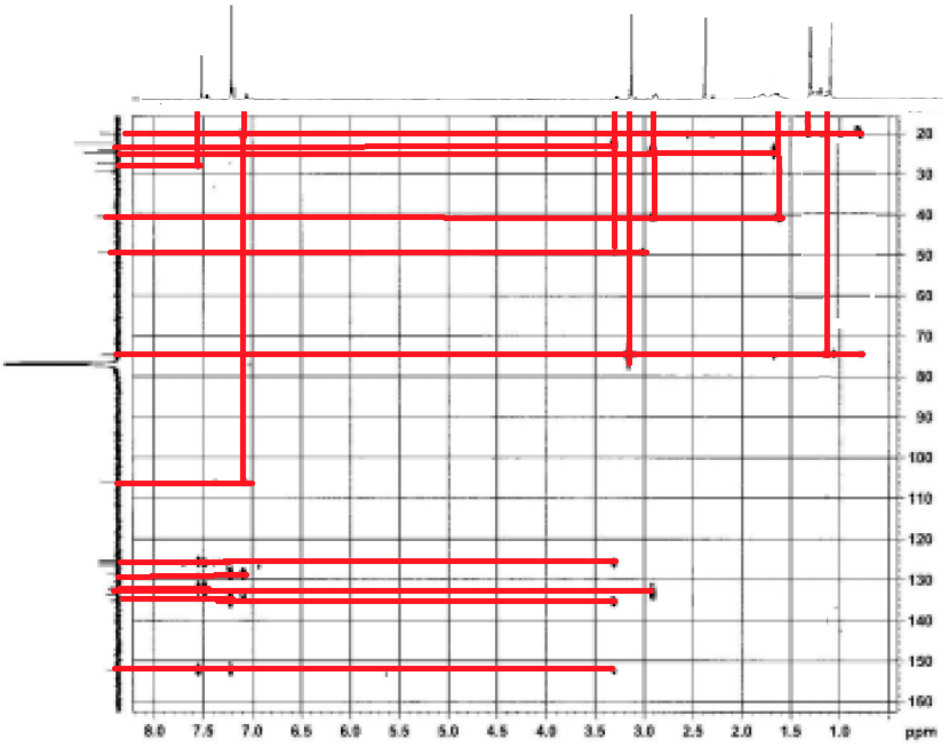

Figure S10. HMBC spectrum of **2**

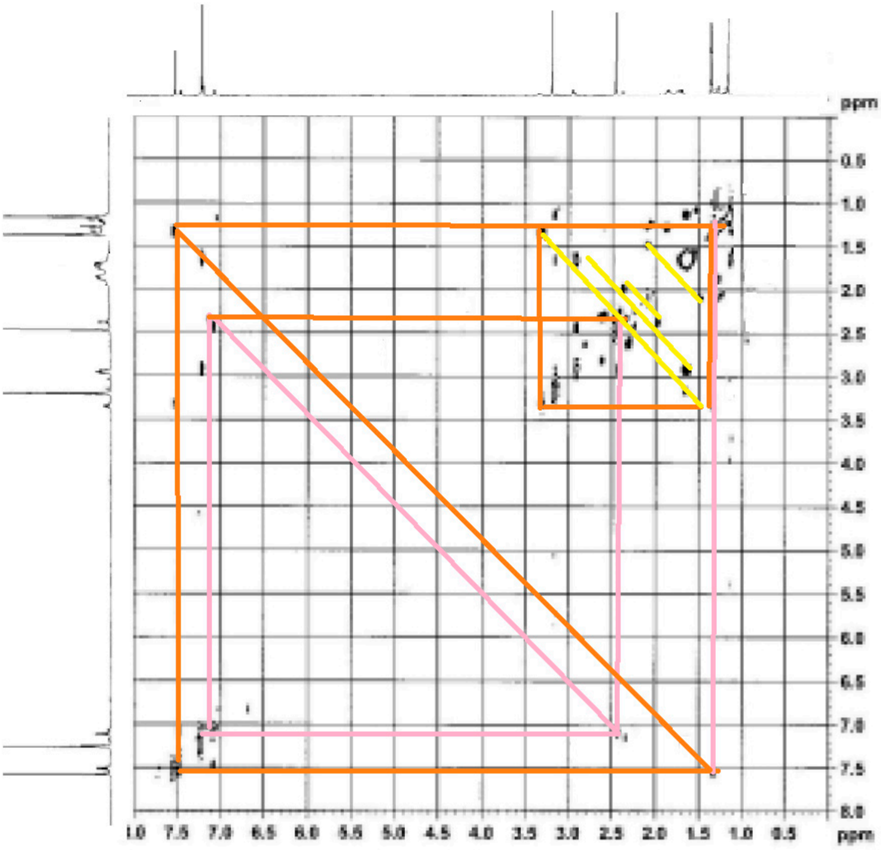

Figure S11. NOESY spectrum of **2**

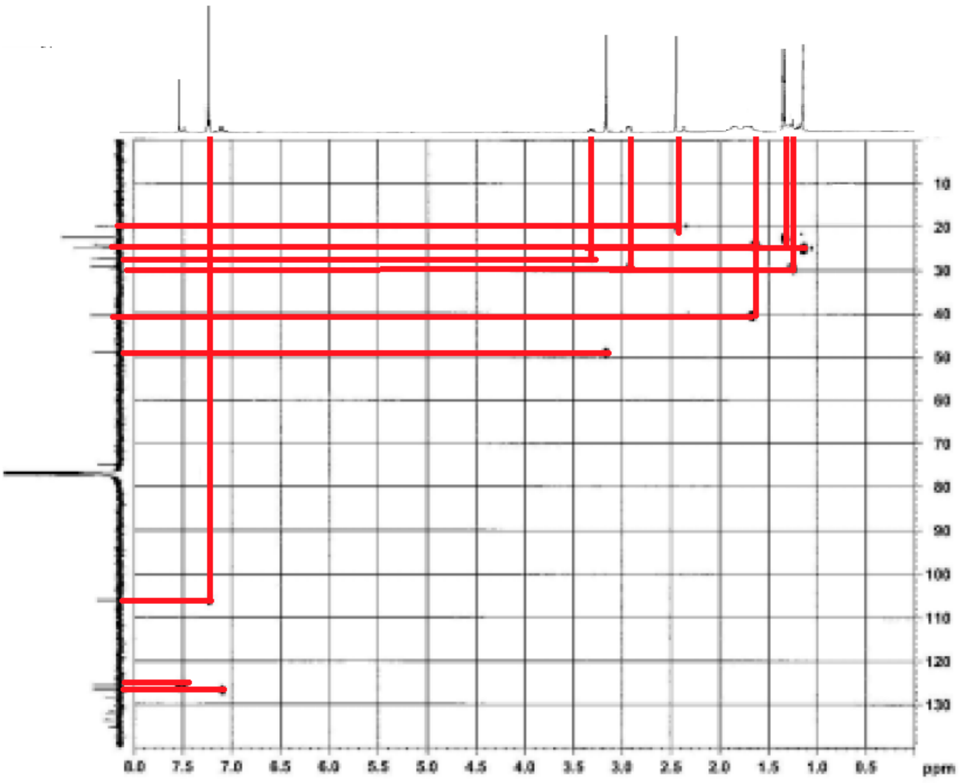

Figure S12. HSQC spectrum of **2**

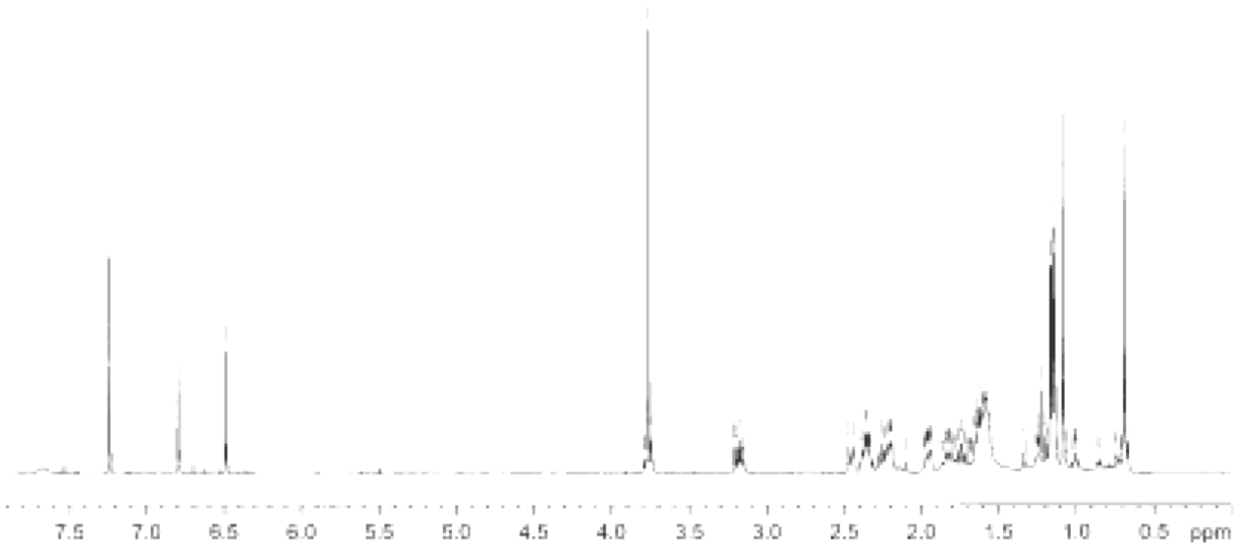

Figure S13. ^{1}H NMR spectrum of **3**

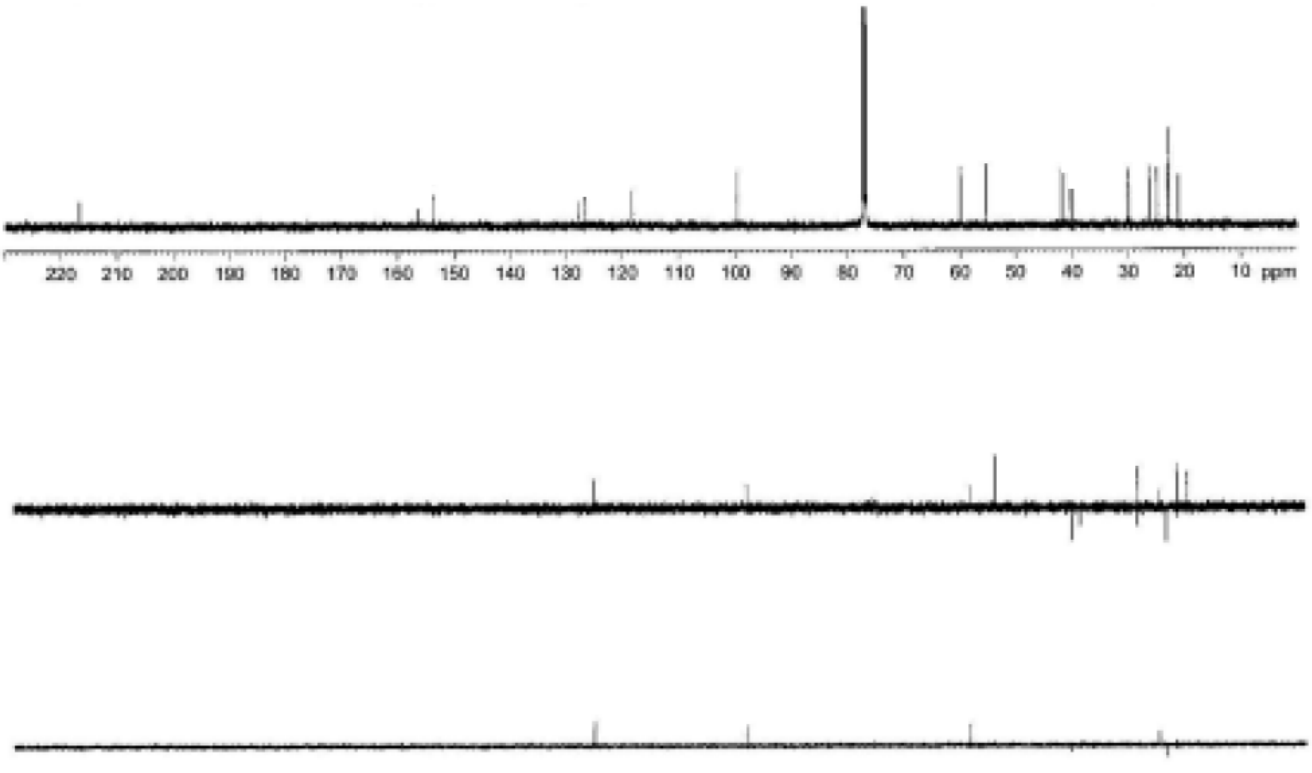

Figure S14. ^{13}C NMR spectrum of **3**

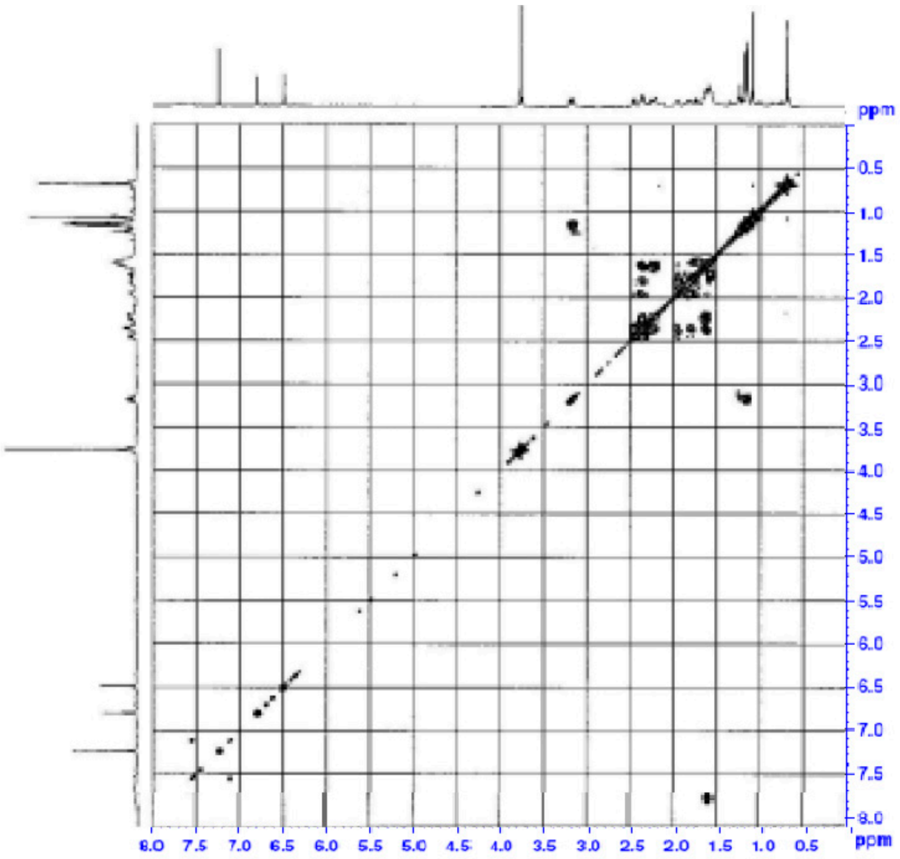

Figure S15. COSY spectrum of **3**

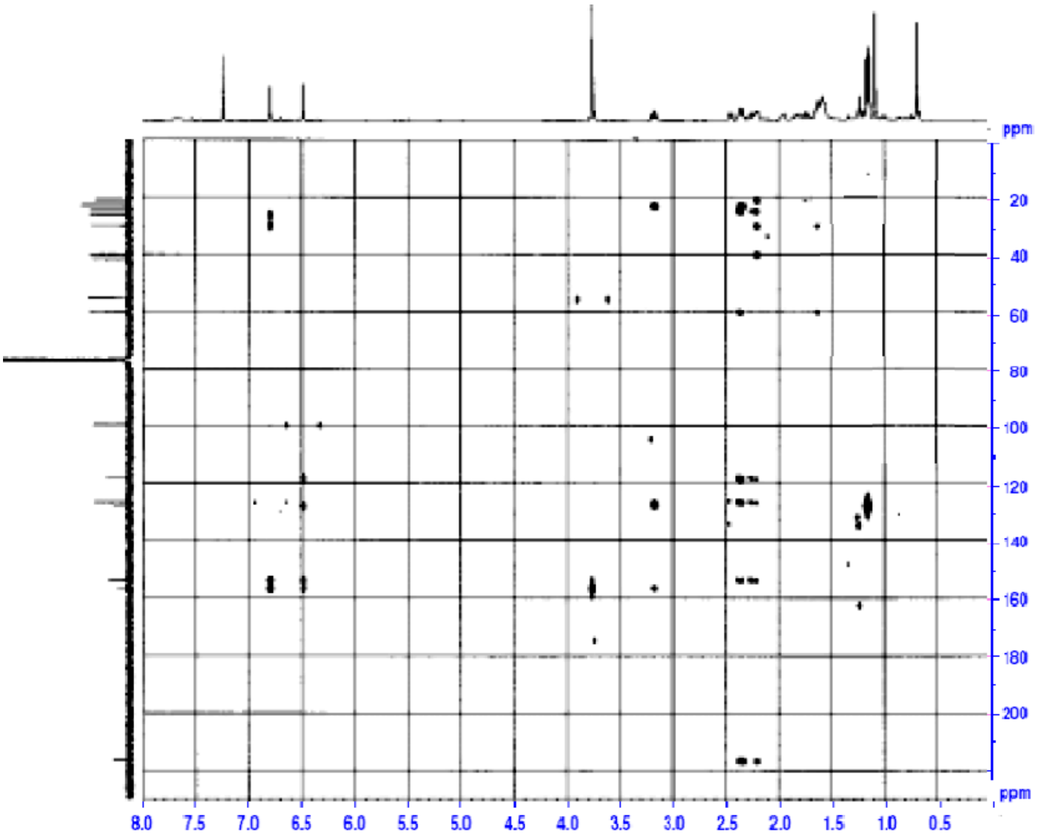

Figure S16. HMBC spectrum of **3**

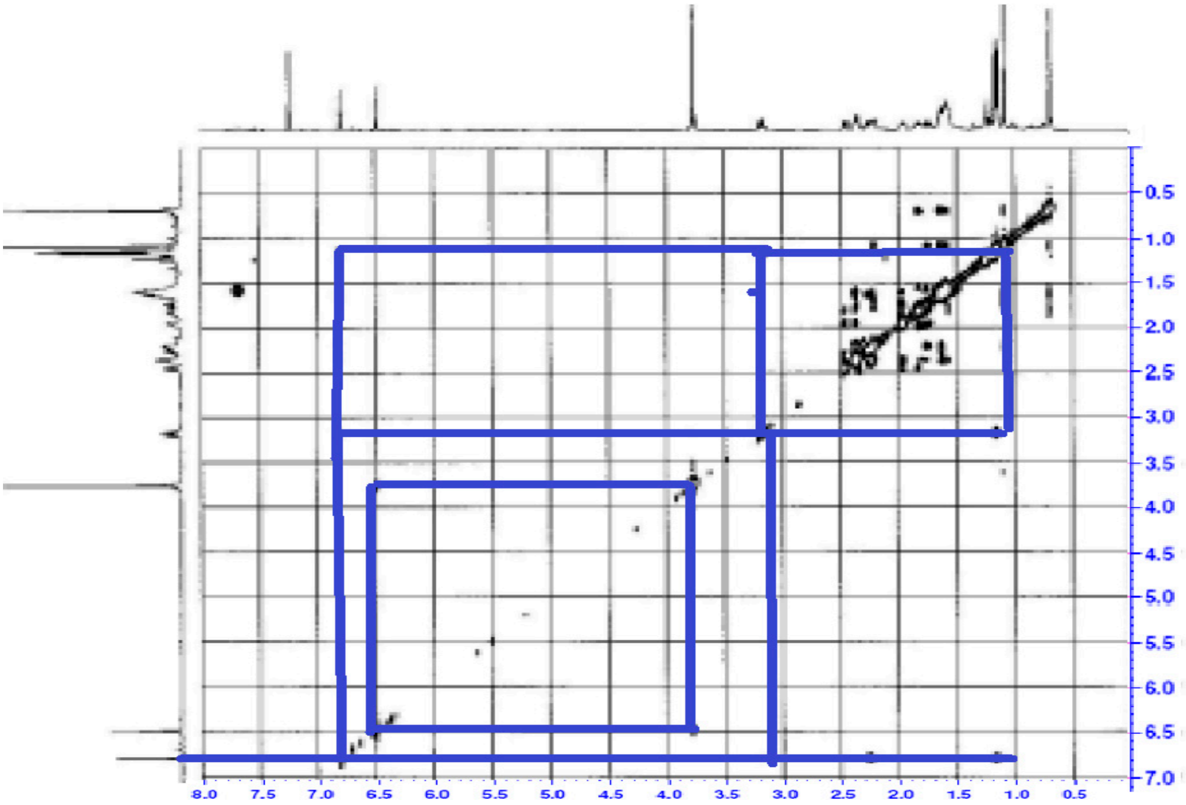

Figure S17. NOESY spectrum of **3**

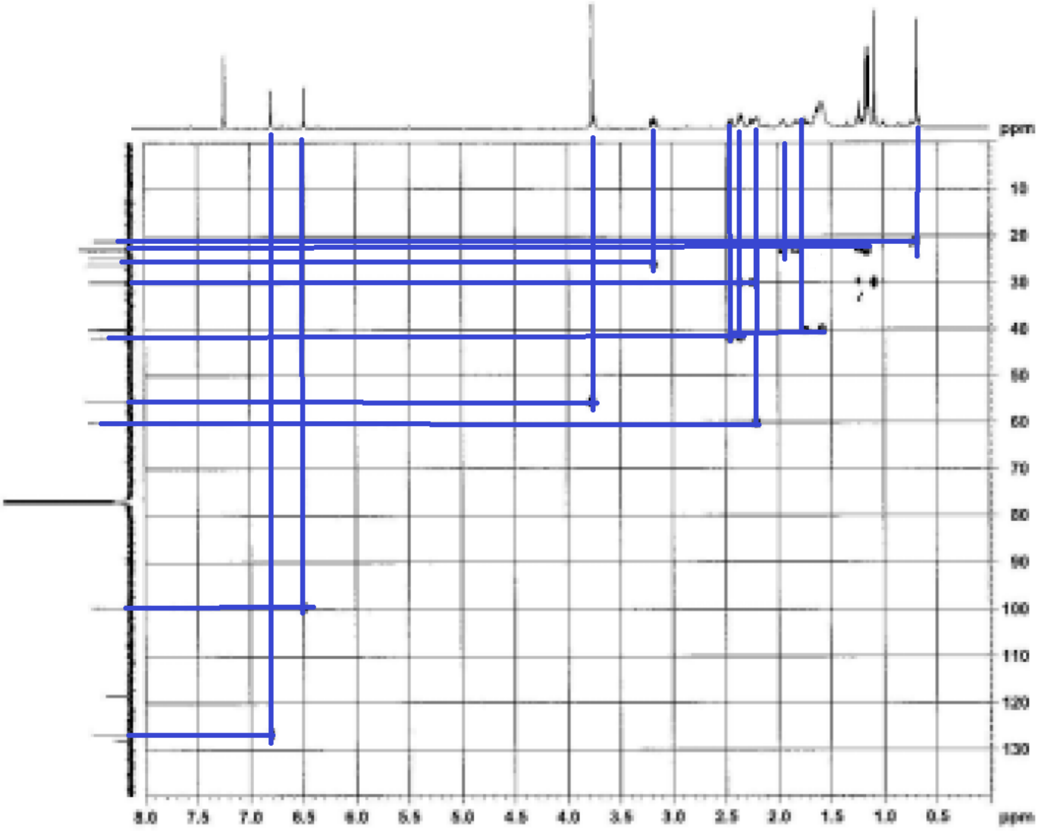

Figure S18. HSQC spectrum of **3**

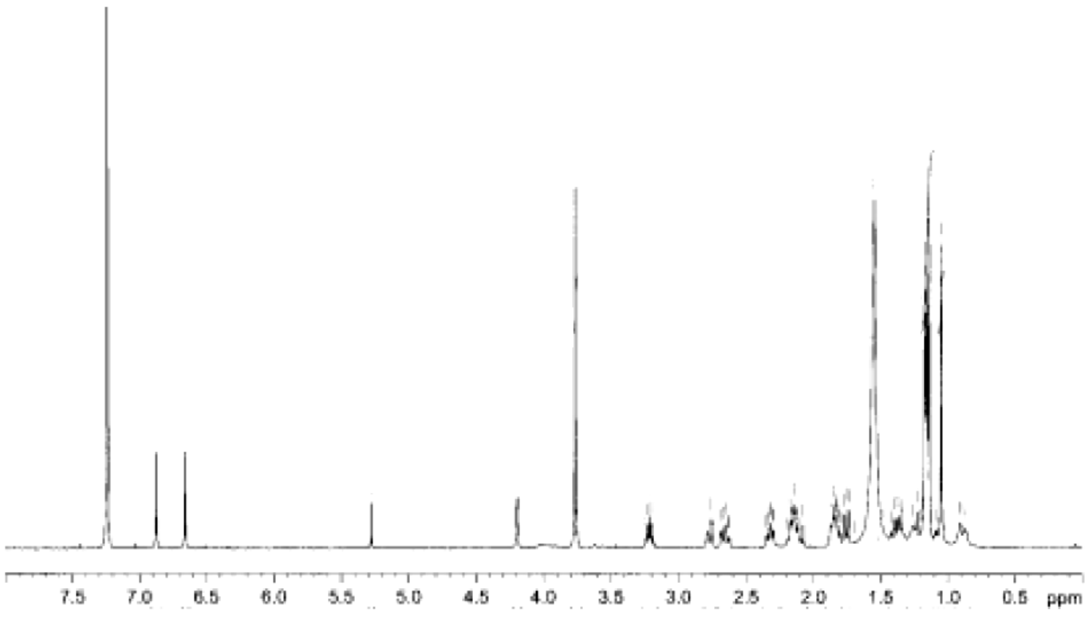

Figure S19. ^{1}H NMR spectrum of **4**

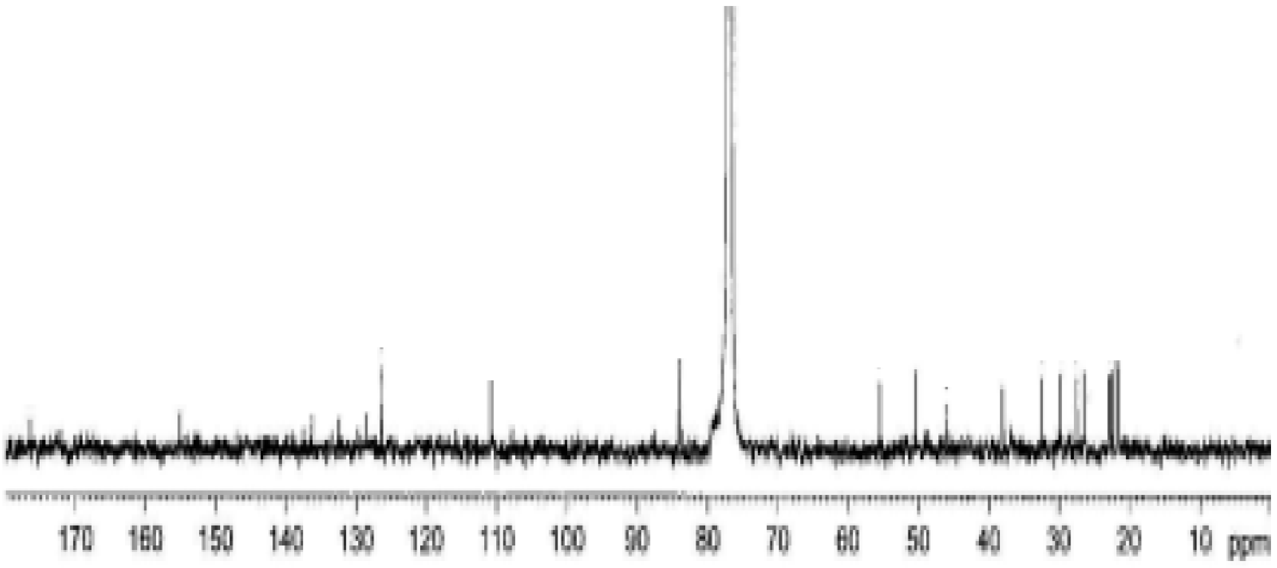

Figure S20. ^{13}C spectrum of **4**

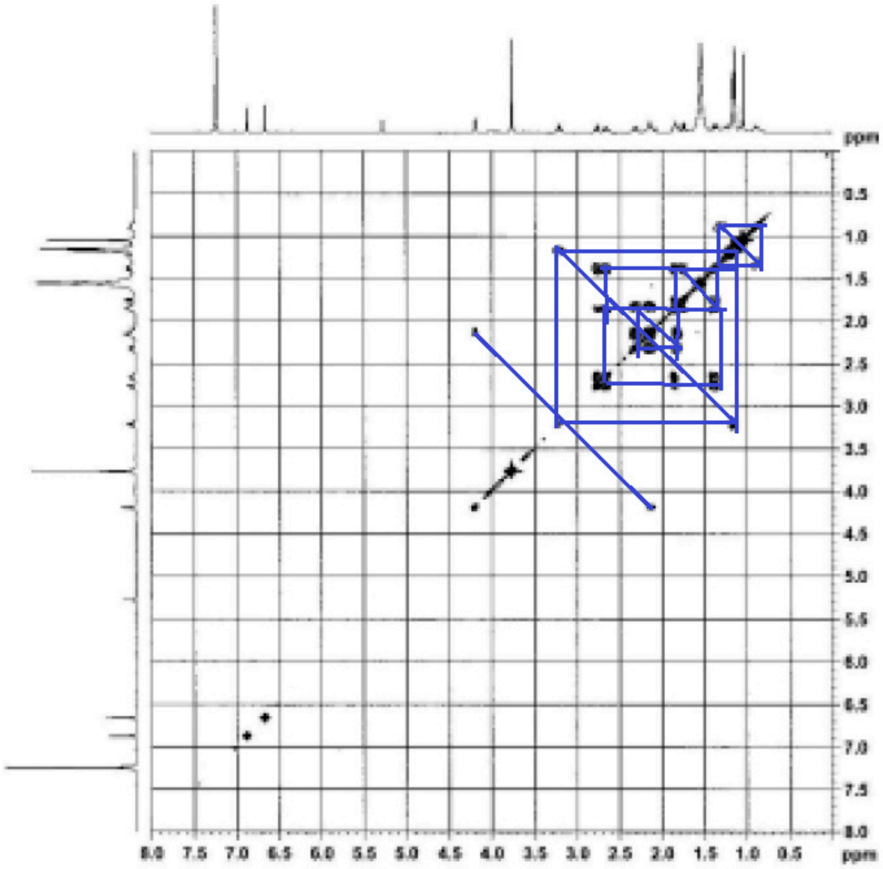

Figure S21. COSY spectrum of **4**

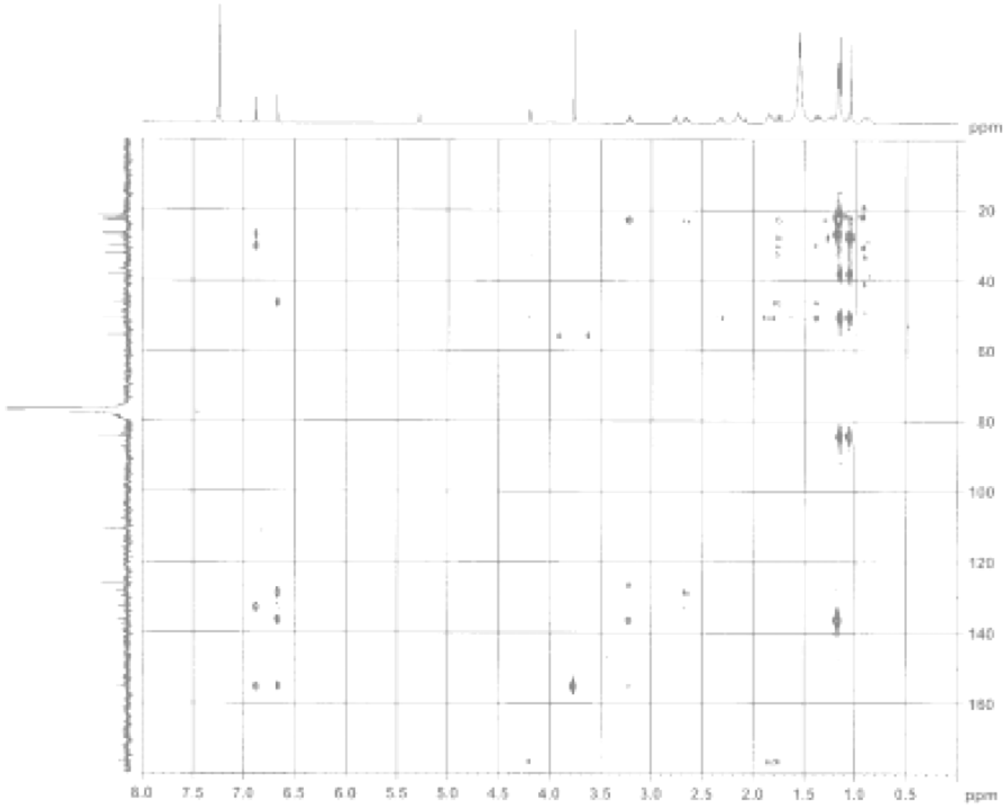

Figure S22. HMBC spectrum of **4**

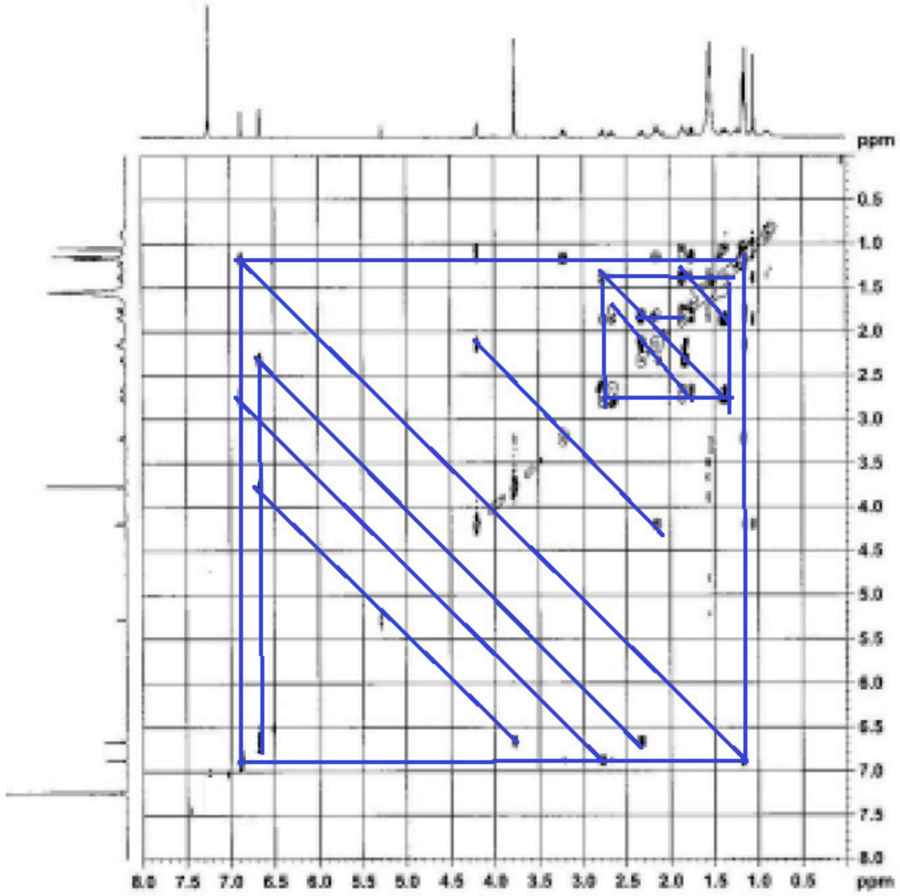

Figure S23. NOESY spectrum of **4**

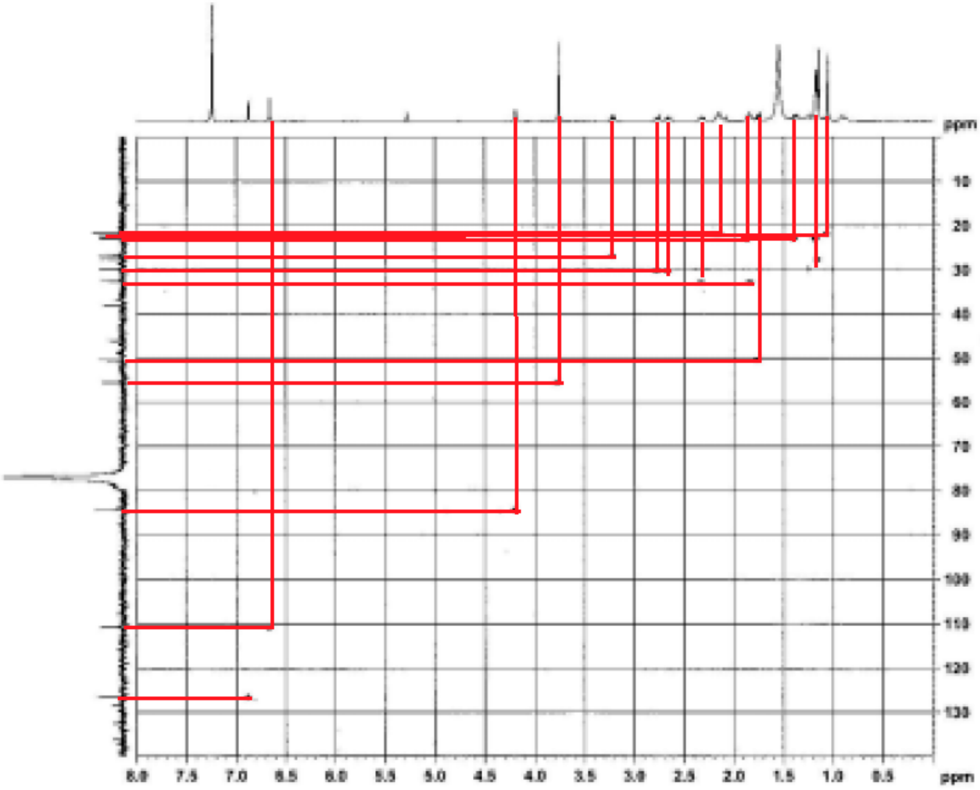

Figure S24. HSQC spectrum of **4**
